# Ecological niche and rickettsial risk mapping of *Dermacentor nuttalli* in Inner Mongolia, China

**DOI:** 10.3389/fcimb.2026.1763405

**Published:** 2026-06-16

**Authors:** Dan Liu, Wulantuya, Yu Wang, Jun-Liang Zhao, Xin Li, Zhi-yuan Lv, Ya-Jie Luo, Ying-jie Wang, Hai-ping Wang, Temure, Hai-cheng Liu, Rui-jun Liu, Rui Zhang, Xia Wang, Jin-Liang Gao, Wen-long Wang, Gaowa

**Affiliations:** 1 College of Veterinary Medicine, Inner Mongolia Agricultural University, Hohhot, China; 2 Inner Mongolia Key Laboratory of Vector-Borne Zoonotic Infectious Diseases, Hetao College, Bayannur, China; 3 Bayannur Animal Husbandry Service Center, Bayannur, China; 4 Bayannur Animal Disease Prevention and Control Center, Bayannur, China; 5 Linhe District Animal Disease Prevention and Control Center, Bayannur, China; 6 Laboratory of Molecular Medicine, Ordos Central Hospital, Ordos, China

**Keywords:** *Dermacentor nuttalli*, ecological niche model, pathogen spectrum, rickettsiae, spatial risk

## Abstract

**Introduction:**

*Dermacentor nuttalli* is the most prevalent and impactful tick species in Inner Mongolia, and serves as an important vector for a variety of pathogenic microorganisms.

**Methods:**

In this study, an optimized ecological niche model was used to analyze the distribution of suitable habitats for *D. nuttalli*. Combined with pathogen detection data from the salivary glands of 8512 adult ticks collected across Inner Mongolia from 2015 to 2025 and relevant literature, we identified 20 tick-associated pathogens. Additionally, we integrated multiple datasets including environmental and ecological factors, vector distribution, host density and transportation-trade indicators to conduct a comprehensive risk assessment of Rickettsiae transmission.

**Results:**

Currently, suitable habitats for *D. nuttalli* cover 56.0% of the total area of Inner Mongolia. The moderate and high suitability areas in Xilingol, Ulanqab and Erdos all account for over 50%. Precipitation of the wettest month (Bio 13) and mean diurnal range (Bio 2) were confirmed as the key driving factors. Under the SSP2-4.5 future climate scenario, the suitable habitat range will continue to expand, with its distribution centroid shifting approximately 134 km southwestward by 2100. Among the detected pathogens, spotted fever group *Rickettsiae* had the highest positive rate and dominated the pathogen community, followed by *Anaplasmataceae* and *Borreliaceae*, which were also frequently reported. Southern Xilingol, northern Ulanqab and northwestern Chifeng were identified as high-risk regions for *Rickettsiae* transmission.

**Discussion:**

This study provides valuable references for the targeted surveillance and prevention of tick-borne rickettsiosis.

## 1 Introduction

*Dermacentor nuttalli*, a tick belonging to the family Ixodidae, is widely distributed across Eastern Siberia, Mongolia, and northern China (Ma H. et al., 2023) and is particularly common in grassland environments suitable for grazing cattle and sheep. Inner Mongolia, which has extensive grassland coverage and shares borders with Russia and Mongolia, is a major livestock husbandry base in China. Its unique natural geographical conditions and large livestock population provide crucial habitats for *D. nuttalli* (Gaowa et al., 2021, 2018). Long-term surveys have shown that from March to June each year, *D. nuttalli* is the main parasitic tick species on livestock in Inner Mongolia, with an extremely wide distribution (Liu et al., 2023; Wulantuya et al., 2021). Additionally, *D. nuttalli* acts as a vector for various zoonotic pathogens; it is capable of carrying and transmitting protozoa, viruses, and bacteria, including *Rickettsia*, posing a significant threat to both public health and animal husbandry (Jiao et al., 2021; Wei et al., 2024). *Rickettsia* spp. are obligate intracellular parasitic pathogens of zoonotic importance, primarily transmitted by ticks and other arthropods. Among them, the spotted fever group and typhus group *Rickettsiae* exhibit significant pathogenicity, causing typical clinical symptoms such as fever and skin rashes in humans; severe cases may involve multiple organ systems. Additionally, these pathogens can establish latent infections in cattle, sheep, small mammals and other animals, forming a zoonotic transmission chain of animal-vector-human (Gui et al., 2022; Černý et al., 2019). We have previously revealed a high spotted fever group *rickettsiae* (SFGR) infection rate in *D. nuttalli*, with an increasing gradient across different ecogeographical zones, including desert, semi-desert steppe, typical steppe, and forest areas (Liu et al., 2023). Environmental variables not only determine the geographical distribution of *D. nuttalli*, but also directly drive pathogens survival, replication and co-feeding transmission efficiency in ticks (Couret et al., 2022; Voyiatzaki et al., 2022). As an ectoparasite, the survival and activity of *D. nuttalli* as well as the processes of pathogen carriage and transmission are influenced substantially by environmental factors (de Souza and Weaver, 2024; Gilbert, 2021; Selmi et al., 2018). Temperature acts as a primary regulator, with activity thresholds varying among tick species (Capligina et al., 2020). Humidity is critical for water balance—the lack of a waxy epicuticle makes ticks highly susceptible to desiccation in arid conditions (Requena-García et al., 2017). Vegetation offers an essential refuge (e.g., on grass blades or in bark crevices). It also attracts host animals, such as rodents and livestock; the diversity and movement of hosts directly shape tick feeding opportunities and dispersal potential (Van et al., 2024; Maldonado-Ruiz et al., 2022). To survive under environmental stresses, such as cold, drought, and prolonged starvation, ticks have evolved various traits, including dormancy, exceptional starvation tolerance, and water regulation mechanisms (Maldonado-Ruiz et al., 2022; Yu et al., 2014). Given the extensive impact of *D. nuttalli* in Inner Mongolia and its strong adaptability to different environmental conditions, systematic analyses of the effect of climate change on the distribution of suitable habitats and transmission risk of pathogens are of key significance for formulating precise prevention and control strategies for ticks and tick-borne diseases, as well as ensuring safe animal husbandry practices.

Ecological niche models (ENMs) are widely used to predict the suitable habitats of species (Inoue and Berg, 2017; de Santana Martins Rodgers et al., 2019; Omar et al., 2021). The Maximum Entropy (MaxEnt) model is based on the theory of species niches (Sofizadeh et al., 2017; Ahmadi et al., 2023). Briefly, a probability distribution is constructed using species occurrence data and environmental background data, this distribution is fitted with maximum entropy values, and the potential distribution of species is estimated. Owing to its advantages, such as its high accuracy, high stability under data-limited conditions, and easy interpretability of results, MaxEnt models have been used to predict tick distributions (Ma R. et al., 2023; Perveen et al., 2024). However, most tick studies using MaxEnt models rely on default parameters for simulations and overlook critical ecological drivers, such as animal host distribution, human activity, and vegetation, potentially leading to model overfitting or underfitting and obscuring accurate identification of potential expansion zones (Ma R. et al., 2023, 2024; Wang et al., 2023, 2024). In addition, current research on tick-borne pathogens still has limitations, focusing mostly on the collation of basic epidemiological data, and further improvement is needed in the systematic quantification and accurate prediction of pathogen transmission risks. Taking the construction of a comprehensive *Rickettsiae* transmission risk prediction model as the entry point, this study further improves the risk assessment system for tick-borne pathogens, which can accurately identify high-risk areas of *Rickettsiae* transmission, and thus provide a reliable scientific basis for the precise monitoring and control of *D. nuttalli* and the early warning of tick-borne rickettsiosis.

## 2 Materials and methods

### 2.1 Ethics statement

The collection of ticks from the body surface of cattle, goats, and horses in this study was verbally approved by the animal owners and performed in strict accordance with the National Guidelines for Experimental Animal Welfare of China (2006-398). In addition, it has been applied and reviewed by the Animal Experimental Ethics Committee of the Medical Department of Hetao College, and it has been put on record in accordance with the “Animal Experimental Ethics Review Measures of Hetao College” (No. HTXY-YXX-2023-003).

### 2.2 Collection of geographical distribution data

A total of 209 distribution records for *D. nuttalli* were compiled from three primary sources. (i) Since 2015, a systematic sampling program has been carried out in the Inner Mongolia region. Over 40,000 samples have been collected, and 134 non-repeated sampling sites were selected. (ii) Using the Figshare database, a review of 251 nationwide GPS records for *D. nuttalli* available as of August 2017 revealed 12 relevant locations within Inner Mongolia. (iii) A literature search was performed. In particular, an extensive search of Chinese databases (CNKI, Wanfang, and VIP) and PubMed using the terms “*Dermacentor nuttalli* OR *D. nuttalli*” AND “Inner Mongolia” generated an additional 63 distribution points (detailed coordinates or described collection sites). The collected records were saved in.xls format and imported into ArcMap 10.2. They were then converted to the Shapefile format and related to the WGS 1984 projection coordinate system. To mitigate data redundancy and potential model overfitting resulting from repeated sampling, the environmental data raster resolution was used as a filter. ENMtools was used to automatically identify and remove redundant records falling within the same grid cell. This process resulted in a final dataset of 153 unique GPS sites for subsequent ecological niche modeling (**Supplementary Table 1** and Supplementary references).

### 2.3 Preparation and screening of bioclimatic environmental variables

#### 2.3.1 Preparation of environmental variables

Data for 19 bioclimatic variables and altitude were sourced from the WorldClim database (http://www.worldclim.org), with the resolution of the variable layers set at 2.5 arc minutes. Livestock density (LSD) data were obtained from the Food and Agriculture Organization of the United Nations (https://www.fao.org/). The soil pH (PH) and soil organic carbon (SOC) content were acquired from the National Earth System Science Data Center (http://www.geodata.cn/). The normalized differential vegetation index (NDVI), population, GDP, land use type (LUT), and other parameters were downloaded from the Resource and Environmental Science and Data Center (https://www.resdc.cn/). Slope and aspect data were generated using the Spatial Analyst tool in ArcMap using the elevation data (**Supplementary Table 2**).

To confine the research area to Inner Mongolia, ArcGIS software tools, such as “Resampling,” “Mask Extraction,” and “Project Raster,” were utilized to extract the point data for bioclimatic environmental variables corresponding to the sampling sites. The geographic coordinate systems and important parameters such as the pixel size (X, Y) of all geographic layers were unified. Finally, the data were converted into ASC format for export for the construction of the MaxEnt species distribution model.

#### 2.3.2 Screening of bioclimatic variables

The distribution records for *D. nuttalli* and all environmental factors were initially loaded into MaxEnt for one-time pre-modeling to acquire the percent contribution and permutation importance of each factor. The jackknife test was used to quantify the contribution ratio and permutation importance of each variable. Variables with both percentage contribution and permutation importance lower than 1% were preliminarily excluded due to weak ecological explanatory power, leaving 23 environmental variables retained after the initial screening. Subsequently, Spearman correlation analyses of the remaining environmental factors were conducted using the ggplot package in R (**Supplementary Figure 1**). For highly correlated variable pairs (|r|s 0.8), the variable with relatively lower percent contribution and permutation importance was removed to minimize multicollinearity. After correlation screening, 16 redundant environmental factors were further removed. Ultimately, seven environmental variables were selected for the MaxEnt model: Mean annual temperature (Bio1), Mean monthly temperature range (Bio2), Isothermality (Bio3), Precipitation of the wettest month (Bio13), Livestock density (LSD), NDVI, and Elevation (ELEV). After completing variable screening, the MaxEnt model was run 10 times using the bootstrapping approach, with a 75% training set, 25% test set, 10,000 background points, 500 iterations, and default values for other parameters (Lan et al., 2022). The final contribution of each environmental factor was quantitatively calculated accordingly.

### 2.4 MaxEnt model optimization and accuracy evaluation

The predictive performance of the model is highly influenced by two key parameters: the regularization multiplier (RM) and feature classes (FC). Therefore, parameter optimization was conducted to mitigate model overfitting and enhance predictive performance. The ENMeval R package (v.3.6.1) was employed to identify the optimal combination of FC and RM. The default parameter settings for the MaxEnt model (RM = 1 and FC = LQHPT) were used as a baseline for comparison. Five feature types were evaluated: linear (L), quadratic (Q), product (P), threshold (T), and hinge (H). Additionally, we increased the RM incrementally by 0.5 within the range of 0.5–4, resulting in 256 parameter combinations. The optimal model was selected based on values for delta AICc (corrected Akaike information criterion), AUC (area under the receiver operating characteristic [ROC] curve), and OR_5_ (5% training omission rate) (Lai et al., 2022).

After removing collinearity among environmental factors, the seven selected variables and spatial occurrence data for *D. nuttalli* were incorporated into the MaxEnt model. The FC and RM parameters were applied, while all other settings remained consistent during model calibration. Model performance was evaluated using the ROC curve and AUC. The AUC value, which ranges from 0 to 1, was used as a threshold-independent measure of prediction accuracy. According to conventional interpretation criteria, AUC values were categorized as follows: <0.7, poor predictive performance; 0.7–0.8, moderate performance; 0.8–0.9, good performance; and 0.9–1.0, excellent performance (Li et al., 2021; Zhu et al., 2017).

### 2.5 Habitat suitability, future projections, and centroid dynamics for *D. nuttalli*

The original logistic output from the MaxEnt model, which represents habitat suitability on a continuous scale from 0 (completely unsuitable) to 1 (fully suitable), was processed to classify the potential distribution of *D. nuttalli*. The ASCII output files were imported into ArcGIS 10.2 and converted into raster format. After visual optimization of the color scheme, the natural breaks (Jenks) classification method was applied to categorize habitat suitability into four distinct grades: No suitability, Low suitability, Medium suitability, and High suitability. In addition, the 10th percentile training presence threshold was used to generate a binary suitability map. Meanwhile, the unclassified continuous suitability surface was retained to intuitively display the gradual change of suitability values from low to high.

To project future distribution shifts, climate data for the periods 2021–2040, 2041–2060, 2061–2080, and 2081–2100 were obtained from the BCC-CSM2.MR model under the CMIP6 framework. For this study, we selected climate change data under the medium-emission SSP2-4.5 scenario (Shared Socioeconomic Pathway 2-Representative Concentration Pathway 4.5), which targets a radiative forcing of 4.5 W/m² by 2100 and aligns with prospective socioeconomic trends in China, making it highly relevant to regional conditions (Wang et al., 2025a). These future climate variables, together with current geographical data and species occurrence records, were incorporated into the pre-optimized MaxEnt model to predict habitat suitability under future scenarios, forming the basis for an ecological early warning system.

The spatiotemporal dynamics of the suitable habitat were further quantified by analyzing the shift in its geographical centroid. The SDM Toolbox was used to calculate the centroid positions for the current and future suitability distributions. The Euclidean migration distance and direction of the centroid were computed using the dists function. This centroid trajectory serves as a synthesized indicator of the overall direction and magnitude of habitat range shift for *D. nuttalli* in response to environmental change.

### 2.6 Associated pathogens and positive rates

#### 2.6.1 Laboratory detection of pathogens

A total of 8,512 adult *D. nuttalli* ticks were collected in Inner Mongolia between 2015 and 2025 (**Supplementary Table 3**). Most ticks were collected from domestic hosts, including cattle, sheep, horses, and camels; therefore, the least engorged specimens were preferentially selected for analysis. A smaller proportion of questing adults were collected from vegetation using the flagging method in forested habitats. Tick species identification was performed by morphological characteristics (Horak et al., 2000) and mitochondrial 16S rDNA (mt-rrs) gene sequencing (Takano et al., 2014).

Live ticks were processed immediately, or preserved at −80 °C until further processing. Ticks were surface-sterilized sequentially with 5% sodium hypochlorite, 75% ethanol, and iodophor for 5 minutes each, then rinsed with sterile water, and air-dried naturally. Under a stereomicroscope, the dorsal cuticle was incised along bilateral body margins with a scalpel to fully expose the internal body cavity. The salivary glands were carefully dissected and collected with caution to avoid contamination from intestinal and other visceral tissues. Genomic DNA was extracted from isolated salivary glands of each individual tick using a commercial DNA extraction kit (Qiagen, Hilden, Germany) according to the manufacturer’s instructions.

For pathogen detection, the citrate synthase (*gltA*) gene of *Rickettsia* was detected by conventional PCR (Gaowa et al., 2018). The outer membrane protein 1 gene (*p28*/*omp1*) of *Ehrlichia* and the major surface protein 2 gene (*p44*/*msp2*) of *Anaplasma* were detected by nested PCR. All PCR assays were performed in a total volume of 25 ul, with 5 ul of template DNA added per reaction. The reaction conditions of the PCR were 3 min at 94 °C, then 35 cycles of 30 s at 94 °C, 1 min annealing at 55 °C, 1 min at 72 °C, and finishing with 10 min at 72 °C. Target DNA fragments of the borrelial flagellin (*flaB*) gene were amplified by conventional PCR as described previously (Kawabata et al., 2001). PCR amplification was performed under conditions of 95 °C denaturation for 30 sec, 50 °C annealing for 30 sec, and 72 °C extension for 60 sec, for a total of 30 cycles. *Babesia* was detected using real-time PCR targeting the 18S rRNA gene (Venter et al., 2024). PCR assays were performed in triplicate, in a total volume of 20 ul, with 2 ul template DNA added per reaction. The reaction conditions of the PCR were 10 min at 95 °C, then 39 cycles of 30 s at 95 °C, 30 s annealing at 57 °C. All primer sequences are listed in **Supplementary Table 4**. Positive PCR products were purified, bidirectionally sequenced (Nanjing Kingsley Biotechnology Company), and confirmed by BLAST alignment with GenBank reference sequences.

#### 2.6.2 Literature data screening and standardization

To complement the laboratory data and comprehensively characterize the pathogen spectrum of *D. nuttalli*, a systematic literature review and meta-analysis were conducted to estimate pooled pathogen prevalence and 95% confidence interval (95% CI). Among all the previously screened literature on *D. nuttalli*, those with the keywords “pathogen” and “prevalence” were further filtered. The inclusion criteria were as follows: studies reporting pathogens associated with *D. nuttalli*, providing explicit prevalence data from the past decade and sample sizes, and adopting PCR pathogen detection methods. The exclusion criteria included duplicate studies, studies with incomplete data, and those with a sample size of less than 10. Core data were extracted, including pathogen name, prevalence, sample size, sampling location, and sampling year. Standardization was performed for pathogen nomenclature and prevalence units, which were uniformly converted to infection rate (%).

#### 2.6.3 Heterogeneity assessment and data integration

When integrating laboratory and literature-derived data, stratified pooled analysis was performed by administrative division. Heterogeneity was assessed using the *I²* statistic: a fixed-effects model was employed when *I²* < 50%, whereas a random-effects model was adopted when *I²* ≥ 50%. For pathogens reported in a single study, prevalence was calculated as the number of positive ticks divided by the total number of tested ticks, without a 95% confidence interval. For those reported in multiple studies, pooled prevalence and 95% confidence intervals were estimated using the meta package in R software (version 4.2.3).

### 2.7 Boosted regression tree model development

Distinct from tick habitat suitability, pathogen transmission involves more complex drivers, and the two belong to entirely independent ecological processes. Therefore, separate modeling of vectors and pathogens was performed to avoid confounding biases. On this basis, the Boosted Regression Tree (BRT) model was adopted to clarify the complex nonlinear relationships between pathogen infection and multidimensional environmental factors. Boosted Regression Tree (BRT) models are often used for ecological niche modeling (Kraemer et al., 2015; Crockett and West, 2014); this approach is similar to logistic regression, with a binary outcome as the dependent variable and environmental variables as the independent variables. A two-stage BRT model was applied to identify potential ecological drivers for the distribution of *Rickettsiae* and to create a high-resolution risk map using the Python packages “sklearn,” “pandas,” and “NumPy” based on locations with two categories of *Rickettsiae* risk. Subsequently, we filtered out variables with a relative contribution of less than 4% through a pre-model for each of the two-stage BRT models by including all of the variables retained after the multicollinearity test. Based on 16 screened variables including 6 eco-climatic variables, land use types, livestock density, population density, aspect, slope, elevation, NDVI, GDP, soil organic content, and soil pH, the stage 1 model was used to distinguish whether there was a risk of *Rickettsiae* occurrence. All locations with medium-low risk, high risk, and unknown risk were classified as “existence” and those with no risk (pseudo-absence records) were classified as “nonexistence.” The stage 2 model further distinguished between high and low-medium risk for areas at risk of *Rickettsiae* occurrence projected by the stage 1 model; the variables included in the second-stage model were largely the same as those in the first-stage model. The key features that contribute the most to the BRT model’s prediction can be identified through the SHAP plot.

### 2.8 *Rickettsiae* integrated risk assessment​

To systematically evaluate the comprehensive risk of *Rickettsiae* in Inner Mongolia, a stepwise risk assessment framework was established involving three sequential steps: occurrence risk assessment, transmission risk assessment, and integrated risk assessment (Supplementary Methods).

Rickettsial occurrence risk reflects the ability of spotted fever group rickettsiae to colonize, persist, and maintain natural circulation in a region, supported by competent vectors, suitable environmental, and sufficient susceptible vertebrate hosts (Liu, 2020). First, a *Rickettsiae* occurrence risk map was generated by spatially overlaying three key indices: climatic suitability, vector suitability, and host density (**Supplementary Figure 2**). Rickettsial transmission risk characterizes the comprehensive probability that rickettsiae are transmitted from infected ticks to susceptible hosts, establish local transmission cycles, and expand geographically, driven by vector abundance, host availability, environmental suitability, and human activity networks (Liu, 2020). Subsequently, a transmission risk map was constructed by incorporating a transportation and trade index, in combination with vector suitability and host density (**Supplementary Figure 3**). For the superimposed approach, the Analytic Hierarchy Process (AHP) method (Saaty, 1994) was employed to assign weights to the risk factors (Supplementary Methods and **Supplementary Tables 5–S7**). The AHP weights were assigned by two experts in tick-borne disease epidemiology and one biostatistician. All three experts hold Ph.D. degrees and senior professional titles, with more than 20 years of relevant research experience. The pairwise comparison matrices were independently constructed by each expert using the 1–9 scale method. The final weights were calculated as the arithmetic mean of individual expert results, and the consistency test (CR < 0.1) confirmed the logical consistency of the judgment matrix. Among all evaluated factors, the vector factors were weighted highest at 0.5. Finally, both risk maps were uniformly classified into low, medium, and high risk levels using the Jenks Natural Breaks Classification method (Gearman and Blinnikov, 2019). Comprehensive *rickettsiae* risk integrates two core components: rickettsial occurrence risk and transmission risk (Liu, 2020). The graded risk maps were further spatially overlaid, with occurrence risk taken as the primary constraint, to generate the final spatial distribution map of comprehensive *rickettsiae* risk (Tran et al., 2016).

Theoretically, this overlay process can yield nine possible risk combinations (e.g., low/low, medium/high, and high/high), allowing the precise identification of regional risk characteristics and providing a scientific basis for developing targeted prevention and control strategies.

## 3 Results

### 3.1 Ecological niche modeling and distribution prediction for *D. nuttalli*

#### 3.1.1 Model optimization and accuracy evaluation

The MaxEnt model was used to forecast suitable locations for *D. nuttalli* under current and future climate scenarios based on 153 occurrence records for the species and 7 screened environmental factors. The optimized FC and RM were QTH and 2, respectively (different from the default parameters of LQHP and 1) (with ΔAICc = 0). As shown in **Table 1**, the AUC value of the optimized MaxEnt model increased from 0.782 with default parameters to 0.811 (the non-repeated AUC was 0.896) (**Supplementary Figure 4A**), and the ΔAICc value decreased from 87.076 to 0, indicating that the model performance was good (**Supplementary Table 8**). The omission rate of the test samples was basically consistent with the prediction omission rate curve (**Supplementary Figure 4B**), indicating that the modeling data did not exhibit spatial autocorrelation and the simulation effect was good. The accuracy of the habitat suitability area of *D. nuttalli* predicted using the MaxEnt model was verified using the on-site sampling data for 2025. The distribution range was all within the potential distribution predicted using the model. Based on a comprehensive evaluation of the AUC values, omission rates, and on-site sampling verification results, the model predictions are highly precise and reliable.

**Table 1:** Prediction of medium and high suitable area of *D. nuttalli* under various environmental variables.

Prefecture-level administrative region	County-level administrative region	Medium and high suitable area	
		Area/km^2^	Ratio/%
Erdos	All County	56,156.2	64.6
Xilingol	All County	12,7391.4	63.7
Ulanqab	All the other counties except for Liangcheng County and Fengzhen City	29,320.9	53.5
Wuhai	Haibowan District	855.8	48.8
Hohhot	Saihan District; Helinger County; Qingshuihe County	7,959.5	45.9
Baotou	Darhan-Muminggan Joint County; Guyang County; Tumed Right Banner;	12,412.2	44.9
BayanNur	Urat Central Banner; Wuyuan County; Urad Front anner; Linhe District	22,331.2	33.7
HulunBuir	New Barag West County; New Barag East County; Prairie Chenbarhu banner; Ergun City; Yakeshi City; Oroqen Autonomous Banner	67,967.1	25.9
Chifeng	Ningcheng County; Harqin Banner; Yuanbaoshan District; Hongshan District; Songshan District; Ongniud Banner	20,714.1	23.0
Tongliao	Naiman Banner	911.1	1.53
Alxa	Alxa East County	1,068.0	0.4
Hinggan	Horqin Right Wing Front Banner	50.1	0.08

#### 3.1.2 Identification of key driving factors

**Figure 1:**
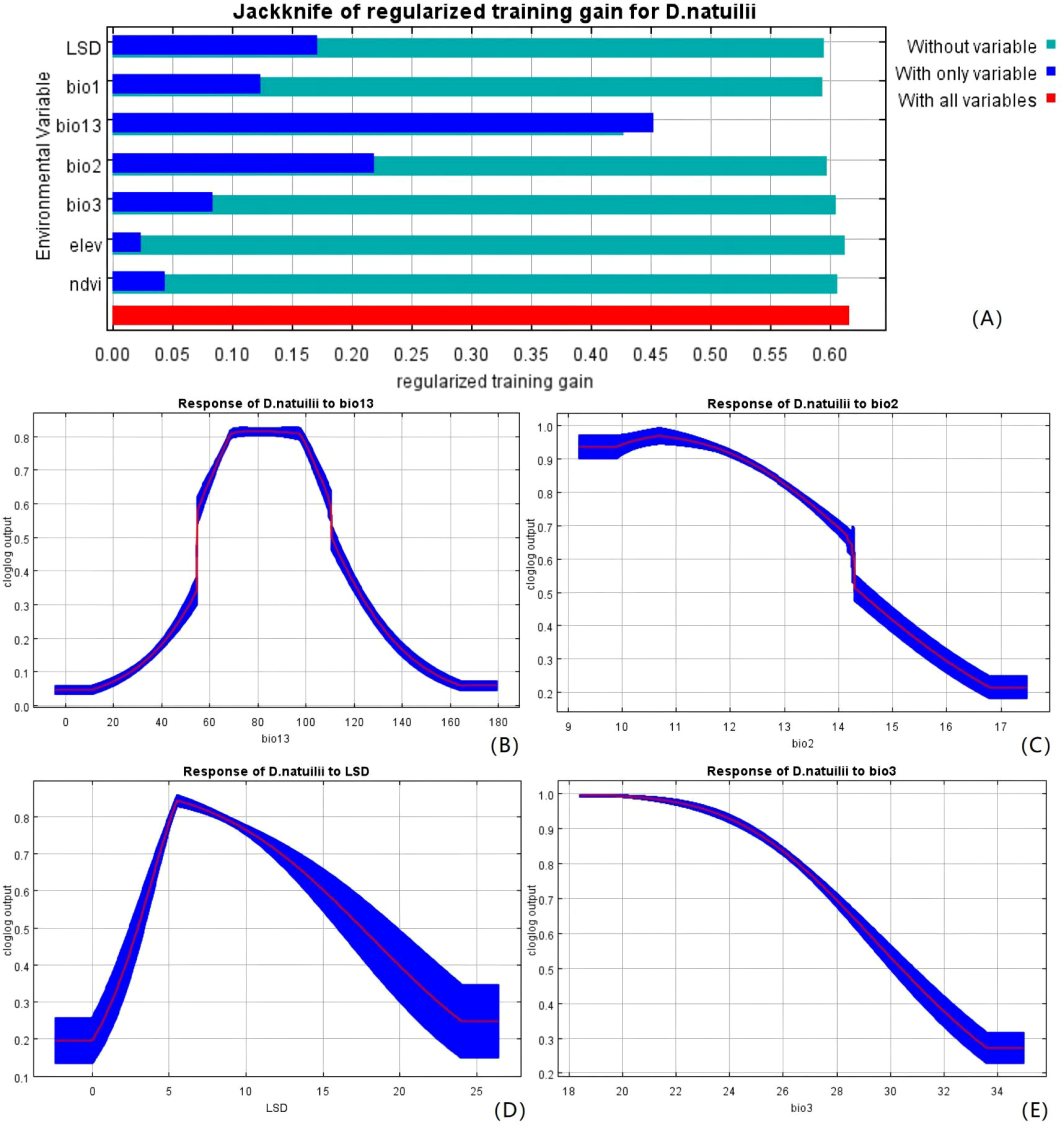
Identification of Key Driving Factors for *D. nuttalli*. **(A)** Jackknife test for a single environmental variable. **(B)** Response curve of Bio 13. **(C)** Response curve of Bio 2. **(D)** Response curve of Livestock density. **(E)** Response curve of Bio 3.

The jackknife method was used to analyze the influence of various environmental meteorological factors on the distribution of potential suitable areas for *D. nuttalli* in Inner Mongolia (**Figure 1A**). When using single-factor modeling, Bio13 and Bio2 had the greatest impact on the prediction model for potential suitable areas of grassland ticks, followed by Bio1 and LSD. If Bio13 was excluded, the gain in model accuracy decreased most significantly; this factor had the greatest impact on the prediction of the distribution of suitable areas. The contribution rates of the seven selected environmental and meteorological factors to the distribution of *D. nuttalli* in Inner Mongolia are shown in **Supplementary Table 9**. Among these, Bio13, Bio2, LSD, NDVI, and Bio3 affected the distribution of *D. nuttalli* significantly, with a percent contribution rate of 97.0% and permutation importance of 85.6%.

Based on the results of the Jackknife test for a single environmental variable as well as the percent contribution and permutation importance, Bio13 and Bio2 were the main factors influencing the potential suitable habitat for grassland ticks. In particular, the most suitable Bio13 for *D. nuttalli* was 88.5 mm, with values ranging from 54.0 to 114 mm remaining suitable (**Figure 1B**). The most suitable Bio2 was 14.7 °C, with values of ≤14.7 °C remaining suitable (**Figure 1C**). The suitable range of LSD values for *D. nuttalli* was 2.1 to 14.9 units (**Figure 1D**). The suitable range of Bio3 for *D. nuttalli* was ≤30 (**Figure 1E**).

#### 3.1.3 Predicted distribution of *D. nuttalli* under current and future SSP2-4.5 climate scenario

**Figure 2:**
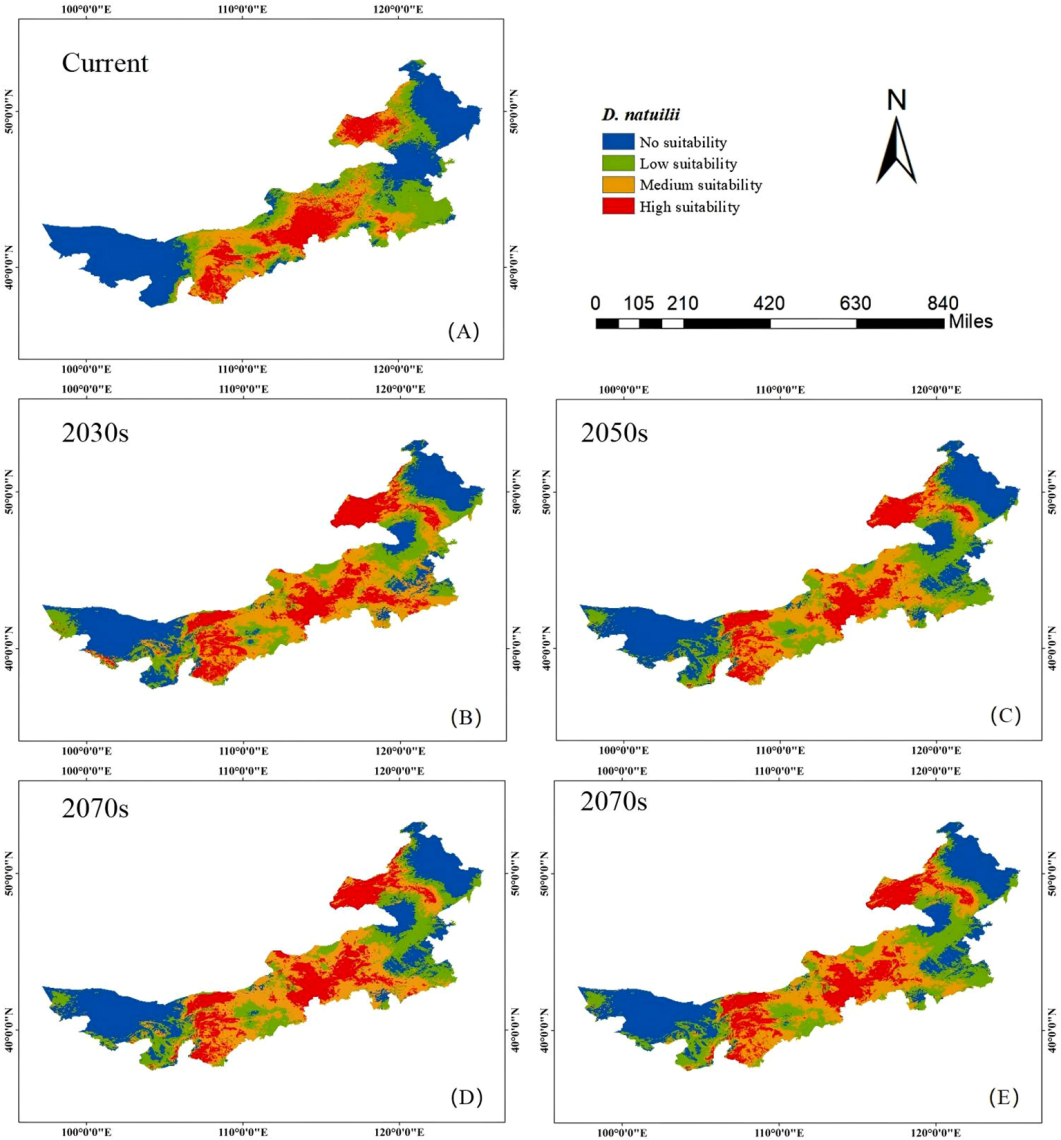
Predicted distribution of *D. nuttalli* under current and future SSP2-4.5 climate scenario. **(A)** Current climate conditions; **(B)** 2030s; **(C)** 2050s; **(D)** 2070s; **(E)** 2070s. Suitability levels are classified as no suitability (blue), low suitability (green), medium suitability (orange), and high suitability (red). Map source: National Earth System Science Data Center (http://www.geodata.cn/data/datadetails.html?dataguid=223718677040067&docid=4590).

The distribution of suitable habitats for *D. nuttalli* in Inner Mongolia exhibited a certain degree of clustering. The total suitable area was 662,000 km^2^, accounting for 56.0% of the total area of Inner Mongolia. The areas of high, medium, and low suitability were 167,000 km^2^, 238,000 km^2^, and 257,000 km^2^, respectively, accounting for 25.2%, 36.0%, and 38.8% of the total suitable area (**Figure 2A**). By superimposing the suitable habitat grid of *D. nuttalli* with the vector map of Inner Mongolia administrative regions and evaluating regional statistics for the medium-high suitable areas, county-level estimates can be obtained (**Table 1**). The medium-high suitable areas of *D. nuttalli* in Xilingol, Ulanqab, and Erdos were all over 50%, ranging from 53.5% to 64.6%. Based on the 10th percentile training threshold, the binary habitat suitability map is shown in **Supplementary Figure 5A**, and the continuous suitability map is shown in **Supplementary Figure 5B**.

Based on the MaxEnt model in the SSP2-4.5 climate scenario, the distribution of potential suitable areas for *D. nuttalli* in Inner Mongolia was predicted for the 2030s, 2050s, 2070s, and 2090s (**Figures 2B–E**). Compared with the current distribution, the potential suitable areas for *D. nuttalli* under future climate scenarios showed no significant shift in geographical position. However, a substantial expansion in area was observed, especially within the medium and high suitability categories. The total suitable area was projected to increase continuously from the current 662,000 km^2^ to 763,000 km^2^ by the end of the century (2081–2100), representing a net growth of 15.2%. This expansion was primarily driven by a remarkable increase in the medium suitability area, which peaked at a 40.3% growth in the near term (2021–2040). The high suitability area also showed significant gains, notably a 31.7% increase in the same period and a further 23.6% rise in 2061–2080. In contrast, the low suitability area exhibited considerable fluctuations, with an initial decrease of 11.3% followed by variable changes in later periods (**Table 2**).

**Table 2:** Change of suitable area of *D. nuttalli* under contemporary and future climate conditions.

Suitable area	Current	2021~2040		2041~2060		2061~2080		2081~2100	
		Area/km^2^	Proportion of change	Area/km^2^	Proportion of change	Area/km^2^	Proportion of change	Area/km^2^	Proportion of change
Total suitable area	662,000	782,000	18.1%	740,000	11.8%	754,000	13.9%	763,000	15.2%
Low suitable area	256,900	228,000	-11.3%	277,000	7.9%	253,000	-1.4%	282,000	9.7%
Medium suitable area	238,000	334,000	40.3%	283,000	19.0%	294,000	23.7%	287,000	20.5%
High suitable area	167,100	220,000	31.7%	180,000	7.4%	207,000	23.6%	194,000	16.3%

**Figure 3:**
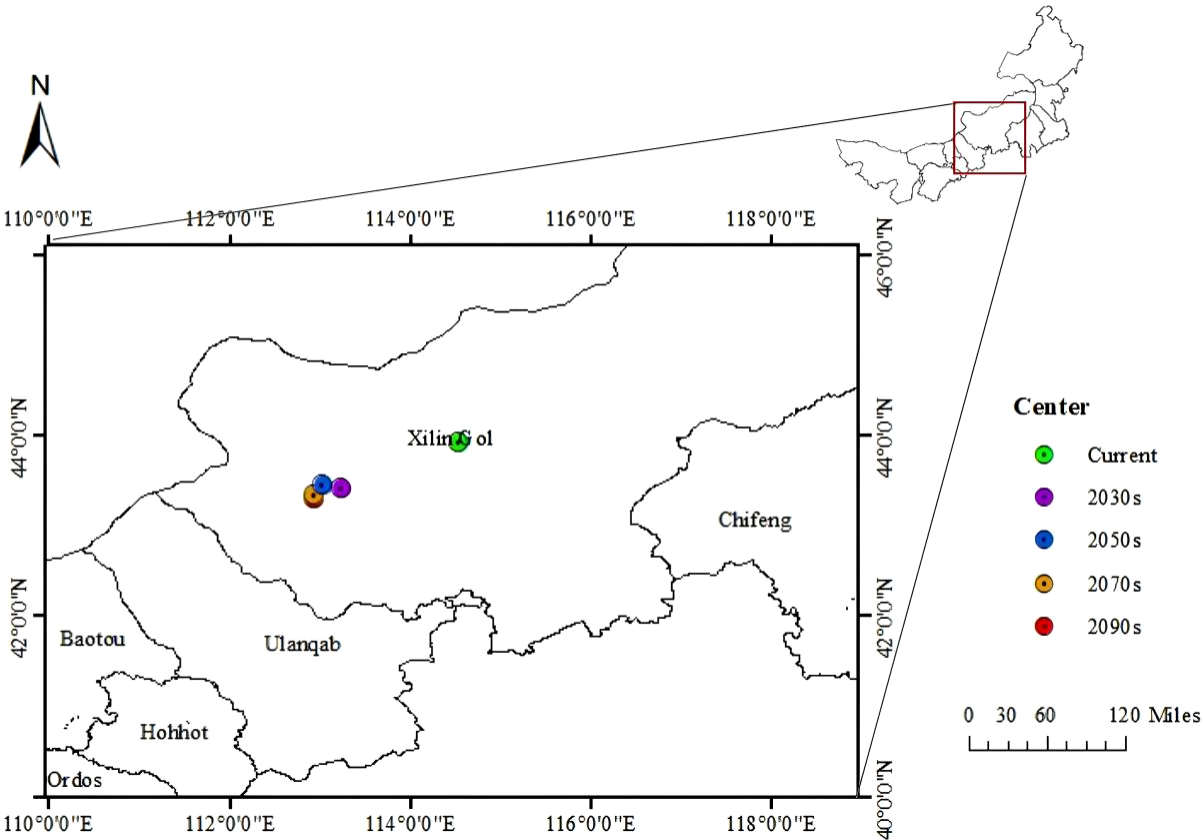
Changes in the centroid of the potential suitability areas for *D. nuttalli* in Inner Mongolia.

The centroid of the potential suitable areas for *D. nuttalli* shifted. By the 2090s, the centroid shifted 134.06 km toward the southwest, with a latitude change of approximately 0.63 degrees and longitude change of approximately 1.60 degrees. The overall potential suitability area also tended to expand toward the southwest (**Figure 3**).

### 3.2 Prevalence of pathogens associated with *D. nuttalli*

Salivary glands were selected for pathogen detection in order to minimize interference from residual host blood present in fed ticks and to reduce the possibility of detecting microorganisms originating from the vertebrate host rather than from the tick itself. This approach enables a more reliable assessment of pathogens potentially maintained and transmitted by *D. nuttalli*. Among the 8,512 adult ticks collected in Inner Mongolia between 2015 and 2025, 4,681 samples were positive for *Rickettsia raoultii* (54.99%), 1,030 for *R. aeschlimannii* (12.10%), 317 for *Rickettsia* sp. YN02 (3.72%), 202 for *Ehrlichia* sp. HF565 (2.37%), 320 for *Borrelia garinii* (3.76%), 194 for *B. afzelii* (2.28%), and 294 for *Babesia caballi* (3.45%).

**Figure 4:**
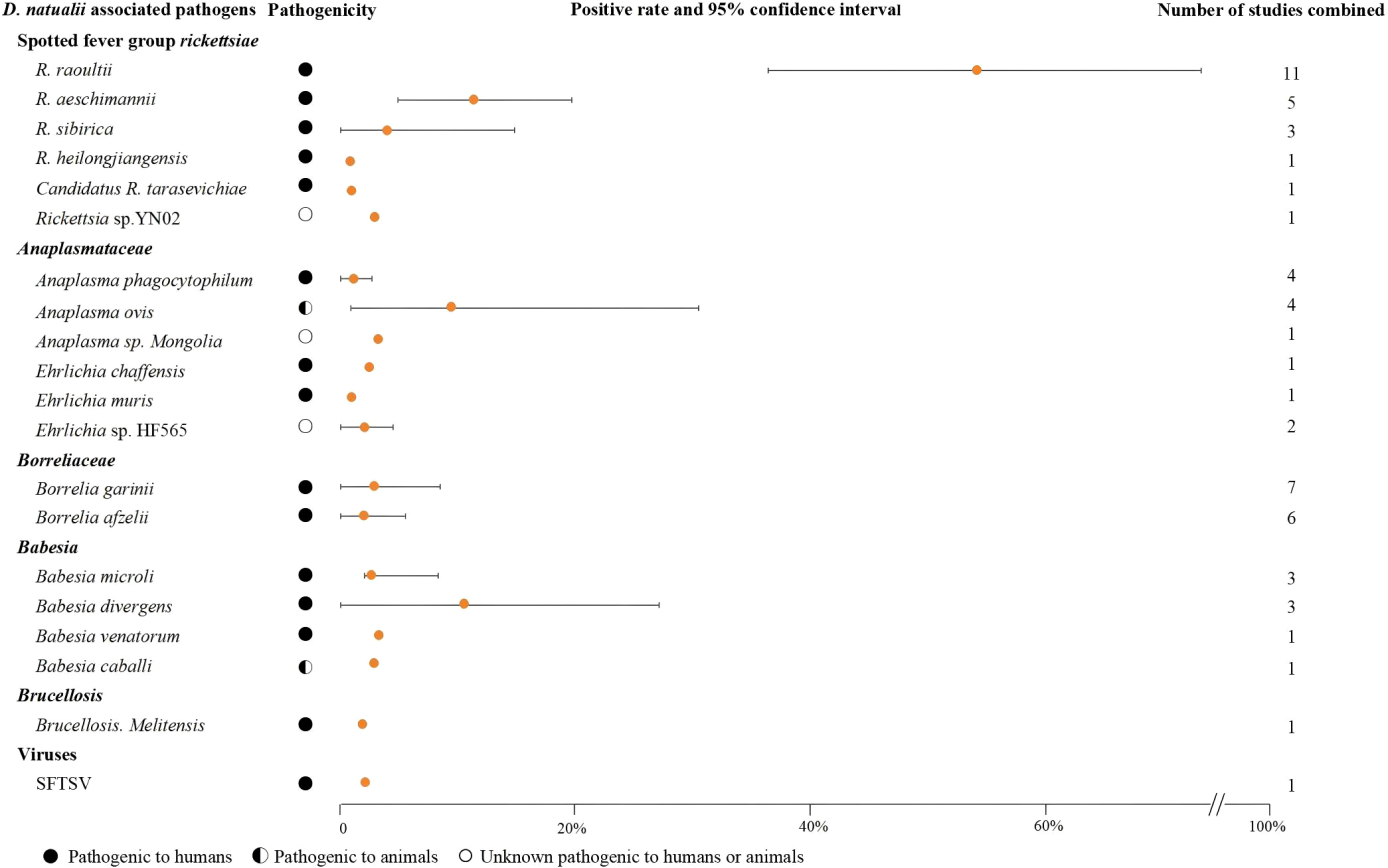
Prevalence of pathogens associated with *D. nuttalli*.

As the *I²* values were all greater than 50%, a random-effects model was applied for data pooling. Based on the integrated analysis, twenty species of microorganisms, including fifteen human pathogens, two animal pathogens, and three agents with unknown risks of pathogenicity were identified in *D. nuttalli* in Inner Mongolia. Six SFGR species were identified in *D. nuttalli*, making it the group with the highest number of species identified among all pathogen categories. All of these SFGR species are human pathogens. Among these species, *R. raoultii* (53.84%, 95% CI 35.82–71.85) and *R. aeschlimannii* (12.19%, 95% CI 4.94–19.44) exhibited high pooled positive detection rates. Six species belonging to the family *Anaplasmataceae* were identified in *D. nuttalli*. Among them, *Anaplasma ovis* had high positive rates (9.67%, 95% CI 1.03–30.0). Two genospecies in the complex Lyme disease *Borrelia* were carried by *D. nuttalli*, among which the pooled positive rates were 3.7% (95% CI 0–8.47) for *B. garinii* and 2.13% (95% CI 0–5.6) for *B. afzelii*. Four species of *Babesia* harbored by *D. nuttalli* were identified, among which *Babesia divergens* had the highest pooled positive rate at 10.98% (95% CI 0–26.7). SFTSV was identified with a positive rate of 2.27%. Other pathogens, such as *Brucellosis melitensis*, was also identified. In conclusion, among all pathogens carried by *D. nuttalli*, *Rickettsiae* poses a higher risk than those of other types of pathogens (**Figure 4**).

### 3.3 Construction of a risk prediction model for *Rickettsiae*

#### 3.3.1 Identification of key driving factors for *Rickettsiae* based on the BRT model

Based on the pathogen detection results, *Rickettsiae* was selected for the construction of a pathogen prediction model, and the performance of BRT model was evaluated (**Supplementary Table 10**). In stage 1 of the BRT model, 11 variables were identified as important drivers for distinguishing whether there was a risk of *Rickettsiae* occurrence (**Table 3**). Three of these variables showed a positive correlation with the occurrence of *Rickettsiae*, among which Elevation has the largest relative contribution (7.3%), followed by Soil organic content and Precipitation seasonality (**Supplementary Figures 6**, **S7**). Population density, soil pH, livestock density, mean diurnal range, aspect, slope, NDVI, and isothermality had nonlinear effects.

**Table 3:** The relative contribution of environmental variables to predict the occurrence risk of *Rickettsiae* based on BRT model.

First stage			Second stage		
Variable	Mean ± sd (%)	Effect	Variable	Mean ± sd (%)	Effect
Population density	15.32 ± 0.22	Nonlinear effects	Temperature seasonality	47.17 ± 2.05	Negative correlation
Soil pH	13.62 ± 0.05	Nonlinear effects	Land use type	17.99 ± 0.68	Nonlinear effects
Livestock density	9.97 ± 0.73	Nonlinear effects	Aspect	10.07 ± 1.50	Nonlinear effects
Elevation	7.29 ± 0.25	Positive correlation	Isothermality	6.47 ± 2.01	Negative correlation
Mean diurnal range	6.62 ± 0.22	Nonlinear effects	Mean diurnal range	4.77 ± 0.68	Nonlinear effects
Soil organic content	6.60 ± 0.60	Positive correlation	Total precipitation	4.64 ± 1.04	Negative correlation
Precipitation seasonality	6.07 ± 0.21	Positive correlation	—	—	—
Aspect	5.24 ± 0.15	Nonlinear effects	—	—	—
Slope	5.07 ± 0.54	Nonlinear effects	—	—	—
NDVI	4.80 ± 0.44	Nonlinear effects	—	—	—
Isothermality	4.10 ± 0.25	Nonlinear effects	—	—	—

In stage 2 of the BRT model, six predictors were showed good performance in discriminating regions at high and low risk of *Rickettsiae* (**Table 3**). Temperature seasonality, isothermality, and total precipitation were negatively associated with the risk of *Rickettsiae*. Nonlinear effects were observed for land use type, aspect, and mean diurnal range. In the stage 2, climatic factors, such as temperature seasonality (47.1%), were dominant factors, followed by environmental and ecological variables, such as Land use type (**Supplementary Figures 8**, **S9**).

#### 3.3.2 Comprehensive risk analysis of occurrence and spread of *Rickettsiae* in Inner Mongolia

**Figure 5:**
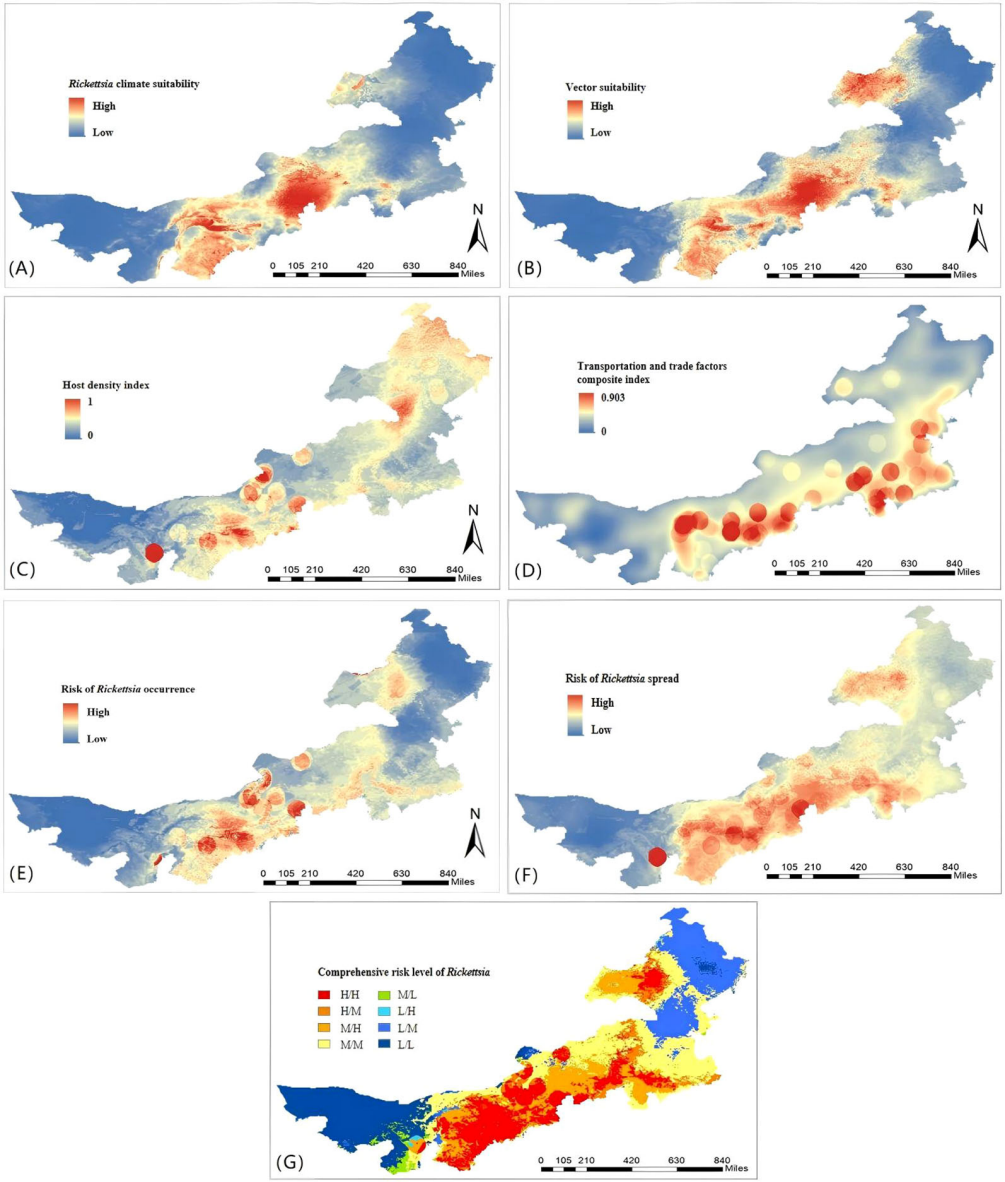
Comprehensive risk analysis of *Rickettsiae* occurrence and spread in Inner Mongolia. **(A)** Map of *Rickettsiae* climate suitability. **(B)** Map of vector suitability (*D. nuttalli*). **(C)** Map of host distribution density index: Obtained through a combined analysis of the population density distribution index (0.1), the livestock density distribution index (0.45), and the rodent density distribution index (0.45). **(D)** Map of transportation and trade factors composite index: Obtained through a combined analysis of the highway density index and the ruminant market density index. **(E)** Risk map of *Rickettsiae* occurrence: Obtained through the fuzzy overlay analysis of *Rickettsiae* climate suitability, vector suitability, and host distribution density index. **(F)** Risk map of *Rickettsiae* spread: Obtained through the combined analysis of host distribution density index (0.3), transportation and trade factors composite index (0.2), and vector suitability (0.5). **(G)** Map of comprehensive risk level of *Rickettsiae*: Obtained by conducting a combined analysis after classifying the risk of occurrence and the risk of spread into three levels: low (L), medium (M), and high (H).

As shown in **Figure 5A**, the areas with high climatic suitability (based on 19 biological and meteorological factors) for *Rickettsiae* were concentrated and distributed in most parts of Xilingol in the central region, northern Ulanqab, and northwestern Chifeng, while areas with low suitability were widely distributed throughout Alxa in the western region and most parts of Hulunbuir in the eastern region. In the central and southern regions with high climatic suitability for *Rickettsiae*, large areas had high suitability for vector distribution (*D. nuttalli*), while the eastern forest areas and western desert areas showed low suitability (**Figure 5B**). As shown in **Figure 5C**, there was significant spatial variation in host density (derived from the weighted integration of livestock, rodent, and population densities) (**Supplementary Figure 10**), with a pronounced east-west gradient and localized clustering characteristics. Specifically, high-density areas were concentrated in the central and northeastern parts of the study region, while low-density zones cover the entire Alxa area in the west, which was predominantly characterized by desert and Gobi landscapes (**Supplementary Figure 10**). **Figure 5D** revealed a distinct spatial concentration of high traffic and trade, quantified by the highway density index and livestock trading market density index (**Supplementary Figure 11**) in the central and eastern parts of the study area. In particular, a contiguous high-value zone was observed, encompassing central Chifeng, the Horqin District of Tongliao, and the southern section of Xilingol. In contrast, low index values were predominantly distributed across the vast western territory of Alxa and forested border areas of Hulunbuir in the north.

The risk assessment maps in **Figures 5E, F** for rickettsial occurrence (superimposed in proportion from **Figures 5A–C**) and transmission (superimposed in proportion from **Figures 5B–D**), respectively, both revealed a highly concentrated high-risk zone in the south-central part of Inner Mongolia. This area specifically encompasses the convergence of southern Xilingol, northern Ulanqab, and northwestern Chifeng. In contrast, low-risk areas were distributed across the entire western region of Alxa and forested areas of the Greater Khingan Mountains in Hulunbuir to the east.

The comprehensive risk level map for *Rickettsiae* shown in **Figure 5G** was obtained by superimposing the risk level map for transmission on the risk level map for occurrence. The High-High risk areas were predominantly concentrated in the central and southern parts of Inner Mongolia, covering the convergence area of southern Xilingol League (including Zhenglan Banner and Taibus Banner), northern Ulanqab (including Siziwang Banner and Chahar Right Middle Banner), and northwestern Chifeng (including Hexigten Banner). The High-Medium and Medium-High risk areas formed a belt-like pattern encircling the High-High risk zone, primarily distributed in the southwestern part of Hulunbuir, northern part of Tongliao, and eastern part of Ordos. In contrast, the Low-Low risk areas were distributed extensively across the entire western region of Alxa and northern forested areas of Hulunbuir in the east.

## 4 Discussion

### 4.1 Optimization and accuracy evaluation of the MaxEnt model

The AICc and the mean OR_5_ of the optimized model decreased, and AUC increased, indicating a significant reduction in model complexity and overfitting, thus improving accuracy. Some authors (Wang et al., 2025b) demonstrated that MaxEnt models with RM = 2.5 and FC = LQP show reduced complexity and overfitting and improved accuracy for predicting the geographical distribution of *Dermacentor everestianus* compared with those for other parameter settings. Huercha found that RM = 1.5 and FC=LQ were the most effective parameters for forecasting the distribution of *D.marginatus* (Huercha, 2021). Some authors determined that the optimal parameters for analyzing the potential fitness zones of *Anaplasma capra* are RM = 2.8 and FC = LQTH (Lin, 2023). These findings suggest that optimal parameter combinations for the MaxEnt model vary among species due to differences in ranges, environmental responses, and biological characteristics.

### 4.2 Dominant environment factors influencing the distribution of *D. nuttalli*

Among the significant influential environmental factors, precipitation was identified as the primary determinant of the *D. nuttalli* distribution, a finding supported by studies of related species, such as *D. silvarum* and *D. everestianus* (Wang et al., 2025; Liu, 2002). Li et al (Li et al., 2022) also clearly identified climate variables, such as temperature and precipitation, as the key factors influencing the distribution of *D. nuttalli*.

​​Precipitation is a core determinant of the geographical distribution of *D. nuttalli*, affecting survival, habitat formation, and host resource acquisition (Li, 2016). Suitable humidity is critical for survival, oviposition, hatching, and molting; arid conditions accelerate water loss and mortality in the species. Moderate rainfall maintains soil and vegetation moisture, creating favorable microhabitats (especially for egg-laying) to support its life cycle (Gern et al., 2025; Bergmann et al., 2025; Zhang et al., 2024). Ample rainfall also promotes lush grasslands, providing more habitats, shelter, and climbing points for ticks to seek hosts (Zhao, 2018). Precipitation indirectly regulates the tick distribution and density by altering the vegetation type and coverage; it also shapes the host (e.g., rodent) population size and distribution via pasture productivity—further constraining tick survival and dispersal.

Temperature is an important determinant of suitable habitats for *D. nuttalli*. As an ectotherm, physiological activities (development, survival, and reproduction) in the species depend heavily on ambient temperature; low temperatures slow metabolism and prolong development, while high temperatures cause heat stress or death. Mean annual temperature, seasonal temperature variation, and extreme temperatures in the coldest/warmest months collectively define the boundaries of its distribution (Li et al., 2022). Temperature also indirectly influences habitat quality and host availability by regulating vegetation growth and host activity. Under climate change, rising temperatures drive the expansion of *D. nuttalli* to higher latitudes and altitudes (Ma R. et al., 2023). Notably, temperature and humidity exert synergistic effects; high temperatures exacerbate drought stress, while suitable humidity mitigates extreme temperature impacts, both jointly shaping the micro-environmental adaptability of *D. nuttalli* (Christoffersen et al., 2024).

### 4.3 Current and future distribution of *D. nuttalli*

Graded classification, binary partitioning and continuous gradient surfaces present habitat suitability characteristics from different dimensions. Jenks grading is conducive to quantitative risk assessment and hierarchical management, binary results simplify regional division, and continuous surfaces retain original model information. These three visualization methods complement one another, which greatly improves the rationality and comprehensiveness of habitat suitability evaluation for *D. nuttalli*.

*D. nuttalli*, a typical grassland tick, is highly dependent on the semi-desert steppe habitat. Its distribution overlaps significantly with that of rodent hosts (e.g., *Citellus dauricus* and *Cricetulus barabensis*), and host-dense regions, such as Xilingol, Ulanqab, and Ordos, reinforce the aggregated distribution of the tick (Liu et al., 2024). These areas, characterized by hilly and sandy steppes, provide diverse microclimates as microhabitat refuges; lush vegetation (30%–60% coverage) maintains conditions with respect to humidity and host availability. Furthermore, traditional Inner Mongolia pastoral areas support high livestock populations, providing ample blood meals for adult ticks, while abundant rodent communities ensure the development of larvae and nymphs, forming a complete host chain for tick persistence.

Model predictions showed that the suitable habitat for *D. nuttalli* in Inner Mongolia will remain stable in the future; however, moderately and highly suitable areas will expand significantly. These results indicate that future climates will enhance current core habitat suitability rather than shift distribution ranges fundamentally, aligning with global warming-driven tick habitat expansion (Ma R. et al., 2023; Kopsco et al., 2023). Similar trends have been observed in Europe, where *D. nuttalli* populations are growing (Springer et al., 2024), and in Heilongjiang Province, where *Ixodes persulcatus* shows expanded suitable habitats under analogous climates (Wang et al., 2024). These expansions may be related to favorable conditions, including rising temperatures and optimized precipitation, in Inner Mongolia under the SSP2-4.5 scenario, especially in Mongolian Plateau transitional zones where temperature and precipitation seasonality will better support *D. nuttalli* survival and reproduction.

Notably, there was a predicted shift in the centroid of suitable habitats for *D. nuttalli* southwestward by approximately 134 km by the end of the 21st century. The westward expansion may be attributed to rising winter temperatures and fewer extreme cold events, transforming previously uninhabitable western regions to viable habitats. This aligns with the identification of temperature and precipitation as core drivers of spatial migration in *Dermacentor*, as confirmed in studies of *D. niveus* and *D. marginatus* (Ma R. et al., 2023; He et al., 2022). Under SSP2-4.5, climate change in western Inner Mongolia may convert unsuitable areas to potential habitats, explaining the westward shift. These changes highlight the need to address an increased tick-borne disease risk from the expansion of moderately and highly suitable habitats and centroid shift, with a particular focus on strengthening monitoring and prevention in southwestern and western grasslands given the preference of *D. nuttalli* for open grasslands.

### 4.4 Prevalence of pathogens associated with *D. nuttalli*

A meta-analysis of pathogens associated with *D. nuttalli* and their global distribution showed that 48 pathogens were identified, including twenty-two human-pathogenic, eight animal-pathogenic, and eighteen with unknown pathogenic risk (Altantogtokh et al., 2022). In the present study, a total of twenty pathogens (fifteen human pathogens) were identified in *D. nuttalli* from Inner Mongolia, confirming the key role of the species as a local tick-borne disease vector. Notably, SFGR species were the most prominent, *R. raoultii* had the highest positive rate, close to the results of molecular epidemiological surveys in Mongolia (> 50%), and higher than its global combined rate of 41.13% in *D. nuttalli* (Wei et al., 2024). This discrepancy may be attributed to the relatively concentrated geographic scope of the present study and the widespread distribution of *D. nuttalli* across different regions of Inner Mongolia.

SFGR was the dominant pathogen carried by *D. nuttalli*. Its high carriage rate indicates a higher probability of pathogen transmission through tick bites (Ma H. et al., 2023). Furthermore, *Rickettsiae* can survive in ticks for a long time and can be transmitted transovarially, further emphasizing the core role of *D. nuttalli* as a transmission vector and reservoir host for *Rickettsiae*.

### 4.5 Construction of a *Rickettsiae* risk prediction model

MaxEnt is well-suited for simulating tick suitable habitats based on presence-only distribution records. In contrast, the BRT model exhibits superior capacity in disentangling complex nonlinear relationships and interactive effects between multiple environmental variables and pathogen transmission risk. Each model possesses unique methodological strengths, and their stepwise application helps to effectively differentiate the distinct driving mechanisms underlying vector spatial distribution and disease transmission. The BRT model was applied to analyze the effects of the environmental variables on the occurrence risk *Rickettsiae*, revealing the potential dynamic driving factors and providing targeted support for *D. nuttalli*-mediated *Rickettsiae* control. A previous study on the global distribution and risk prediction of SFGR compared multiple prediction models to explore the ecological drivers underlying their distribution, and finally confirmed that the BRT model had the optimal comprehensive performance (Zhang et al., 2023), which identified annual mean temperature, precipitation, and host density as key influencing factors. In the present study, temperature seasonality was the dominant factor and was negatively correlated with *Rickettsiae* positive rates. A study modeling the distribution of *R. japonica* in China and Asian adjacent regions using the Maxent model also demonstrated that temperature seasonality and precipitation emerged as pivotal climatic factors influencing the potential distribution of *R. japonica* (Wang et al., 2025a). This may be closely related to the growth and developmental rhythms of *Rickettsiae* and its vector *D. nuttalli*, as well as the seasonal temperature fluctuations affecting tick activity and pathogen reproduction, with stronger seasonality reducing *Rickettsiae* prevalence (Lalzar et al., 2012).

Risk assessment maps for *Rickettsiae* occurrence and transmission consistently indicated a highly concentrated high-risk area in south-central Inner Mongolia, specifically covering the junction of southern Xilingol, northern Ulanqab, and northwestern Chifeng. As a traditional core livestock husbandry region in Inner Mongolia, the risk distribution pattern in this area is highly consistent with the characteristics of high livestock density, intensive grazing activities, frequent cross-regional livestock trade and circulation, as well as favorable climatic and vegetation conditions for tick survival and reproduction. In contrast, low-risk areas were widely distributed across western Alxa and the forested regions of the Greater Khingan Mountains in eastern Hulunbuir. For large-scale animal disease transmission, transportation and trade play crucial roles, alongside host and vector factors (Boyang, 2020). Southern Inner Mongolia borders provinces including Hebei, Shanxi, Shaanxi, and Ningxia, with convenient transportation networks with the potential to facilitate long-distance and large-scale disease spread.

Regions with high occurrence risks showed substantial overlap with regions with high transmission risks. Specifically, High-High risk areas were predominantly concentrated in south-central Inner Mongolia, including the convergence zone of southern Xilingol League, northern Ulanqab, and northwestern Chifeng. This finding suggests these regions face a high risk of localized *Rickettsiae* outbreaks and an elevated risk of epidemic spread without effective, targeted prevention and control measures. Consequently, focused surveillance and prevention efforts are necessary in these areas to address potential *Rickettsiae*-related risks.

## 5 Limitations

This study has several limitations that must be considered when interpreting results, especially risk modeling outcomes, as they constrain the validity and scope of conclusions. First, sampling bias may exist, as sites were selected based on accessibility and prior *D. nuttalli* distribution records, which may underestimate the representativeness of remote area and affect the accuracy of the assessment of the risk of *Rickettsia* transmission. Second, it should be emphasized that this study integrated published pathogen detection data with our own results to analyze the pathogen spectrum of *D. nuttalli*. However, data integration has certain limitations, including inconsistent detection methods and sample tissues, as well as potential differences in sample sources and sampling periods across studies, which may affect the comparability of the results. Third, this study focused on livestock hosts, while other wildlife hosts are also important *D. nuttalli* hosts and key to *Rickettsia* transmission, were not incorporated into the MaxEnt model, leading to an incomplete understanding of the transmission system. Fourth, model uncertainty is unavoidable: despite optimized MaxEnt and BRT models were used for prediction, they rely on existing data, such as sample bias, parameter settings, environmental variables selection, and expert ratings differences may affect model stability and predictability. Thus, the high-risk regions identified by the models should be treated with discernment in line with the study’s limitations. These limitations guide future research to improve study reliability and comprehensiveness.

## 6 Conclusion and prospect

This study innovatively integrates niche models and machine learning algorithms to establish a preliminary comprehensive risk assessment framework for tick-borne pathogens, providing a reference for identifying potential high-risk regions. Based on available data and optimized models, potential high-risk areas for *Rickettsiae* transmission in Inner Mongolia were identified, mainly including the border zones of southern Xilingol, northern Ulanqab, and northwestern Chifeng, Ordos, and future medium-to-high suitable habitats for *D. nuttalli*. These are preliminary predictions, however, affected by sampling bias, single-time-point detection, and model uncertainty, and cannot be regarded as definitive risk distributions. To address the inferred westward expansion of *D. nuttalli* habitat, targeted monitoring in western region should be strengthened to supplement remote area data. Veterinarily, standardized acaricides use in key areas, and enhanced cross-regional livestock quarantine are recommended, with dynamic adjustments as more comprehensive data are obtained. Public health efforts should include tick bite prevention and rickettsiosis symptoms training for herdsmen and outdoor workers in potential high-risk areas, noting actual risk may vary due to unaccounted factors. Future research should address current limitations by incorporating grazing management and livestock trade scale into models, conducting long-term multi-time-point detection across all seasons, expanding sampling to remote areas and wildlife hosts, and verifying key sites to optimize prevention measures and improve risk assessment reliability.

## Data Availability

The original contributions presented in the study are included in the article/**Supplementary Material**. Further inquiries can be directed to the corresponding authors.
