# Supplementary material for: Ecological niche and rickettsial risk mapping of Dermacentor nuttalli in Inner Mongolia, China
Source: Front Cell Infect Microbiol. 2026 Jun 16;16:1763405. doi: 10.3389/fcimb.2026.1763405 (PMC13314851; doi:10.3389/fcimb.2026.1763405)
Supplement: Supplementary file 1 [file DataSheet1.pdf]

Supplementary Material

Table S1. Location information for *D. nuttalli*

number	longitude	latitude	lc_level	lc_l1	town_ID	Name	lc_l3	lc_l4	lc_l5	host	Year	Source
1	108.92	40.63	5	Inner Mongolia	9	Bayan Nur	Wulate Qianqi	Baiyanhua	Wulanbaolige	sheep	2015	Sampling
26	108.48	38.81	4	Inner Mongolia	8	Ordos	Etuokeqi	Sumitu	—	sheep	2015	Sampling
32	106.84	39.71	3	Inner Mongolia	12	Wuhai	Moergou	—	—	sheep	2015	Sampling
33	121.11	48.57	4	Inner Mongolia	3	Hulunbuir	Yakeshi	Mianduhe	—	forest	2016	Sampling
34	121.39	48.43	4	Inner Mongolia	3	Hulunbuir	Yakeshi	Zhuoyuan	—	forest	2017	Sampling
37	114.96	44.02	3	Inner Mongolia	6	Xilin Gol	Abaga	—	—	sheep	2017	Sampling
62	115.99	42.24	4	Inner Mongolia	6	Xilin Gol	Lanqi	Shangdu	—	cattle	2017	Sampling
63	120.13	50.55	4	Inner Mongolia	3	Hulunbuir	Yakeshi	Mianduhe	Jiaojie	forest	2018	Sampling
65	115.35	44.47	4	Inner Mongolia	3	Hulunbuir	Abagaqi	Alatan	—	sheep	2018	Sampling
78	112.90	42.32	4	Inner Mongolia	6	Xilin Gol	Sunitezuoqi	Duhumu	—	sheep	2018	Sampling
79	114.50	43.12	5	Inner Mongolia	6	Xilin Gol	Sunitezuoqi	Bayan	Eerdun	sheep	2018	Sampling
93	120.09	43.87	3	Inner Mongolia	2	Chifeng	Alukeerqin	—	—	sheep	2019	Sampling
94	108.08	39.23	4	Inner Mongolia	8	Ordos	Etuokeqi	Chahannaoer	—	sheep	2019	Sampling
133	121.70	44.04	5	Inner Mongolia	5	Tongliao	Keerqin	Zuoyizhongqi	Xibohua	sheep	2019	Sampling
134	119.79	48.01	4	Inner Mongolia	3	Hulunbuir	Xinbaerhuzuoqi	Wubuerbaolige	—	sheep	2019	Sampling
100	111.95	40.92	3	Inner Mongolia	1	Hohhot	Xincheng	Baoheshao	S105	sheep	2021	Sampling
101	107.22	41.19	5	Inner Mongolia	9	Bayan Nur	Wulate Zhongqi	Wugai	Bayan	goat	2021	Sampling
107	111.26	42.68	4	Inner Mongolia	7	Ulanqab	Siziwang Banner	Herimenwusu	—	sheep	2021	Sampling
108	108.27	38.91	5	Inner Mongolia	8	Ordos	Etuokeqi	Sumitu	Xihaotu	sheep	2023	Sampling
115	106.66	41.29	5	Inner Mongolia	9	Bayan Nur	Wulatehouqi	Huogeqi	ganqimaodu	goat	2023	Sampling
123	108.537777	41.441131	4	Inner Mongolia	9	Bayan Nur	Wulatezhongqi	Tonghetai	—	sheep	2023	Sampling

128	105.66190	38.83296	4	Inner Mongolia	10	Alxa	Zuoqi	Bayanhaotezhen	—	—	—	https://info.figshare.com/
130	111.96618	43.41778	4	Inner Mongolia	6	Xilin Gol	Erlianhaote	Gerileaodu	—	—	—	https://info.figshare.com/
131	117.60369	49.50003	4	Inner Mongolia	3	Hulunbuir	Zhalainuoer	Disijiedao	—	—	—	https://info.figshare.com/
144	120.074059	43.880192	4	Inner Mongolia	2	Chifeng	Alukeerqinqi	Tianshan	—	sheep	2021	CNKI
145	108.318437	37.703826	3	Inner Mongolia	8	Ordos	Etuokeqianqi	chenchuanzhen	—	sheep	2021	CNKI
146	112.650296	42.747579	3	Inner Mongolia	6	Xilin Gol	Sunitezuoqi	—	—	goat	2023	CNKI
152	119.701677	44.728391	5	Inner Mongolia	2	Chifeng	Alukeerqinqi	Tianshan	Bayanwendu	sheep	2018-2020	Wanfang
153	120.112219	47.061611	4	Inner Mongolia	4	Hinggan	Arxan	Bailang	—	sheep	2018-2020	Wanfang

Supplementary references for the sources of geographical information for *D. nuttalli*

[1] Man Da, Ao Xiaofang, Celemuge, et al. Isolation and identification of' a tick-derived isolate of *Enterococcus faecalis* and its pathogenicity in mice. Heilongjiang Animal Science and Veterinary Medicine. 2024.(11):67-71+120. (in Chinese)

[2] Cui Hao. A Comparative Study on the Microbial Community Structure and Diversity Of *Dermacentor nuttalli* at Different Growth Stages in Two Regions of Inner Mongolia[D]. Inner Mongolia Agricultural University,2024. (in Chinese)

[3] Wu Youhong. Systematic classification of *Dermacentor* based on morphological characteristics and mitochondrial genomes[D]. Hebei Normal University,2024. (in Chinese)

[4] Cui Mengyu. Spatial Distribution of Ticks and Genetic Diversity of Important Tick Species in Inner Mongolia[D]. Inner Mongolia Medical University,2024. (in Chinese)

[5] Yang Junqi, Jiao Wanming, Ma yuan, et al. Study on the killing effect of entomogenous fungi Metarhizium brunneum on *Dermacentor nuttalli*[J]. Heilongjiang Animal Science and Veterinary Medicine,2023,(21):26-30+124.. (in Chinese)

[6] Ma Yimin, Zhou Wenkai, Yang Bo, et al. Identification, Artificial Feeding and Life History Observation of *Dermacentor nuttalli* in Inner Mongolia[J]. Northwest Journal of Agricultural Sciences,2023,32(06):835-843.

[7] Xing Lili. Establishment of Triple Fluorescence Quantitative PCR Methodfor Detection of Tick-borne *Rickettsia* Disease [D]. Inner Mongolia Medical University,2023. (in Chinese)

[8] Ma Yimin. Artificial breeding and microbial community composition of *Dermacentor nuttalli* in Inner Mongolia[D].Inner Mongolia Agricultural University,2022. (in Chinese)

[9] Huang Tianpeng, Guoxu, Sun Changyun, et al. Isolation and Identification of *Brucella melitensis* from Ticks on Cattle Surface in Hulunbuir Area[J].[9] Agricultural Sciences of China,2022,55(02):415-424. (in Chinese)

[10] Huang Tianpeng. Study on the Traceability of *Brucella Melitensis* from Inner Mongolia and the Characteristics of Its New Arthropod Host [D].Inner Mongolia Medical University,2020. (in Chinese)

[11] Chaomulige, Eerdunbaoligao, Tianpeng Huang, et al. Isolation and identification of a tick-borne salt and alkali tolerant bacterium *Oceanobacillus oncorhynchi* IMH [J]. Acta Microbiology,2019,59(05):841-850.

[12] Fan Yuanyuan. Detection of Rickettsia in *Dermacentor nuttalli* and the effecton host genetic diversity[D].Hebei Normal University,2019.

[13] Jiang Lu, Xu Liangui, Zhu Jinguo, et al. The first detection of *Coxiella burnetii* in *Dermacentor nuttalli* in Argun port[J]. Chinese Journal of Frontier Health and Quarantine,2017,40(02):108-109+112.

[14] Jiang Zaijie. Morphological Study of Nymphs of grassland ticks and forest ticks [J]. Journal of Beijing Normal University (Natural Science Edition),1987,(01):68-75.

[15] Huang Fang. Genetic Diversity of Dominant Ticks and Detection of Specific Genes of *Brucella* in Xilingol League, Inner Mongolia.

[16] Gui Z, et al. Identification and genetic diversity analysis of *Rickettsia* in *Dermacentor nuttalli* within inner Mongolia, China. Parasites Vectors. 2022;15:286. doi: 10.1186/s13071-022-05387-4.

[17] Zhao L, Ma YM, Yang B, Han WX, Zhao WH, Chai HL, Zhang ZS, Zhan YJ, Wang LF,

Xing Y, Yu LF, Wang JL, Ding YL, Liu YH. Comparative analysis of microbial communities in different growth stages of *Dermacentor nuttalli*. Front Vet Sci. 2022 Oct 14;9:1021426. doi: 10.3389/fvets.2022.1021426.

[18] Jiao J, Lu Z, Yu Y, Ou Y, Fu M, Zhao Y, Wu N, Zhao M, Liu Y, Sun Y, Wen B, Zhou D, Yuan Q, Xiong X. Identification of tick-borne pathogens by metagenomic next-generation sequencing in *Dermacentor nuttalli* and *Ixodes persulcatus* in Inner Mongolia, China. Parasit Vectors. 2021 May 27;14(1):287. doi: 10.1186/s13071-021-04740-3.

[19] Su S, Cui MY, Xing LL, Gao RJ, Mu L, Hong M, Guo QQ, Ren H, Yu JF, Si XY, Eerde M. Metatranscriptomic analysis reveals the diversity of RNA viruses in ticks in Inner Mongolia, China. PLoS Negl Trop Dis. 2024 Dec 11;18(12):e0012706. doi: 10.1371/journal.pntd.0012706.

[20] Kong Y, et al. Phylogenetic analysis of Crimean-Congo hemorrhagic fever virus in inner Mongolia, China. Ticks Tick-Borne Dis. 2022;13:101856. doi: 10.1016/j.ttbdis.2021.101856.

[21] Kong Y, et al. Metatranscriptomics reveals the diversity of the tick virome in Northwest China. Microbiol Spectr. 2022;10:e01115–e1122. doi: 10.1128/spectrum.01115-22.

[22] Su S, Hong M, Cui MY, Gui Z, Ma SF, Wu L, Xing LL, Mu L, Yu JF, Fu SY, Gao RJ, Qi DD. Microbial diversity of ticks and a novel typhus group *Rickettsia* species (Rickettsiales bacterium Ac37b) in Inner Mongolia, China. Parasite. 2023;30:58. doi: 10.1051/parasite/2023057.

[23] Yin X, Guo S, Ding C, Cao M, Kawabata H, Sato K, Ando S, Fujita H, Kawamori F, Su H, Shimada M, Shimamura Y, Masuda S, Ohashi N. Spotted Fever Group *Rickettsiae* in Inner Mongolia, China, 2015-2016. Emerg Infect Dis. 2018 Nov;24(11):2105-2107. doi: 10.3201/eid2411.162094.

[24] Lv J, Wu S, Zhang Y, Chen Y, Feng C, Yuan X, Jia G, Deng J, Wang C, Wang Q, Mei L, Lin X. Assessment of four DNA fragments (COI, 16S rDNA, ITS2, 12S rDNA) for species identification of the Ixodida (Acari: Ixodida). Parasit Vectors. 2014 Mar 3;7:93. doi: 10.1186/1756-3305-7-93.

[25] Huang T, Zhang J, Sun C, Liu Z, He H, Wu J, Geriletu. A Novel Arthropod Host of Brucellosis in the Arid Steppe Ecosystem. Front Vet Sci. 2020 Oct 23;7:566253. doi: 10.3389/fvets.2020.566253.

[26] Fan M, et al. Rickettsial and serologic evidence for prevalent spotted fever rickettsiosis in inner Mongolia. Am J Trop Med Hyg. 1987;36:615–620. doi: 10.4269/ajtmh.1987.36.615.

[27] Liu Q-H, Chen G-Y, Jin Y, et al. Evidence for a high prevalence of spotted fever group rickettsial infections in diverse ecologic zones of Inner Mongolia. Epidemiology and Infection. 1995;115(1):177-183. doi:10.1017/S0950268800058246.

[28] Hao G-F, et al. Detection of tick and tick-borne pathogen in some ports of Inner Mongolia. Zhonghua liu Xing Bing xue za zhi. 2009;30:365–367.

[29] Zhu J-G, Xu L-G, Chen M-C, et al. Tick investigation at Erguna port and adjacent forest area in 2015. Chin J Front Health Quar. 2016;39:3.

[30] Jin Y, Te M, Liu Q-H, et al. Isolation and identification of *Rickettsia* Ha-91 strain from Inner Mongolia-Amun spotted fever group. Chin J Zoonoses. 1993;9:3.

[31] Duan D-Y, Zhou H-M, Cheng T-Y. Effects of Blood sucking Behavior on the Microbial Community in the Midgut of *Dermacentor nuttalli* and *Dermacentor silvarum.* Acta Veterinaria et lootechnica Sinica 2020; 51(01): 128-36.

[32] Li Y-N, Chen Y-F, Yun T-Y. Dominant species of ticks and tick-borne pathogens in the central and western ports areas of the Inner Mongolia Autonomous Region, China, 2020. Chin J Vector Biol & Control, 2022, 33(2): 216-220.

[33] Yang Y, Gao Y-F, Cao Y, et al. Investigation on tick-borne pathogens in Inner Mongolia Manchuria port area during 2012-2014. Chin J Vector Biol & Control, 2018, 29(2): 147-150, 156.

[34] Wei H, Xiong T, Wang SS, Wang BH, Du LF, Xu Q, Zheng JJ, Cui XM, Jia N, Jiang JF, Shi W, Zhao L, Cao WC. Investigating the pathogens associated with *Dermacentor nuttalli* and its global distribution: A study integrating metagenomic sequencing, meta-analysis and niche modeling. Int J Parasitol Parasites Wildl. 2024 Jan 16;23:100907. doi: 10.1016/j.ijppaw.2024.100907.

Table S2. Description of 29 potential influencing factors included in model development

Variable	Description	Source	Data period	Resolution
Bio1	Annual average temperature (℃)	https://www.worldclim.org	1970-2000	2.5 min of arc
Bio2	Mean diurnal range (Mean of monthly max - min temperature) (℃)	https://www.worldclim.org	1970-2000	2.5 min of arc
Bio3	Isothermality (BIO02 ÷ BIO07 ×100) (%)	https://www.worldclim.org	1970-2000	2.5 min of arc
Bio4	Temperature seasonality (standard deviation of mean temperature over 12 months of a year)	https://www.worldclim.org	1970-2000	2.5 min of arc
Bio5	Max temperature of warmest month (℃)	https://www.worldclim.org	1970-2000	2.5 min of arc
Bio6	Min temperature of coldest month (℃)	https://www.worldclim.org	1970-2000	2.5 min of arc
Bio7	Annual range of temperature (BIO05-BIO06) (℃)	https://www.worldclim.org	1970-2000	2.5 min of arc
Bio8	Mean temperature of wettest quarter (℃)	https://www.worldclim.org	1970-2000	2.5 min of arc
Bio9	Mean temperature of driest quarter (℃)	https://www.worldclim.org	1970-2000	2.5 min of arc
Bio10	Mean temperature of warmest quarter (℃)	https://www.worldclim.org	1970-2000	2.5 min of arc
Bio11	Mean temperature of coldest quarter (℃)	https://www.worldclim.org	1970-2000	2.5 min of arc
Bio12	Annual precipitation (mm)	https://www.worldclim.org	1970-2000	2.5 min of arc
Bio13	Precipitation of wettest month (mm)	https://www.worldclim.org	1970-2000	2.5 min of arc
Bio14	Precipitation of driest month (mm)	https://www.worldclim.org	1970-2000	2.5 min of arc
Bio15	Precipitation seasonality (coefficient of variation in rainfall over 12 months of the year)	https://www.worldclim.org	1970-2000	2.5 min of arc
Bio16	Precipitation of wettest quarter (mm)	https://www.worldclim.org	1970-2000	2.5 min of arc
Bio17	Precipitation of driest quarter (mm)	https://www.worldclim.org	1970-2000	2.5 min of arc
Bio18	Precipitation of warmest quarter (mm)	https://www.worldclim.org	1970-2000	2.5 min of arc
Bio19	Precipitation of coldest quarter (mm)	https://www.worldclim.org	1970-2000	2.5 min of arc
ELEV	Average altitude (m)	https://www.worldclim.org	1970-2000	2.5 min of arc
POP	Population density	http://www.resdc.cn/	2020	1 km
GDP	Gross domestic product	http://www.resdc.cn/	2020	1 km
NDVI	Normalized Difference vegetation Index	http://www.resdc.cn/	2023	1 km
LUT	Land use type	http://www.resdc.cn/	2020	1 km
PH	Soil PH	http://www.geodata.cn/	2022	250 m
SOC	Soil organic carbon content	http://www.geodata.cn/	2022	250 m
Slope	Inclination degree of a specific surface point	Calculated from the Elevation	1970-2000	2.5 min of arc
Aspect	Geographical orientation of slope aspect	Calculated from the Elevation	1970-2000	2.5 min of arc
LSD	Livestock density (1 head per km²)	https://www.fao.org/faolex/zh/ (Including the density of cattle, sheep, and goat)	2010	5 min of arc

Figure S1. Correlation matrix for 29 variables.

Heatmap representing the correlations between features using Pearson correlation coefficients. Red and blue in the plot represent positive and negative linear relationships, respectively.

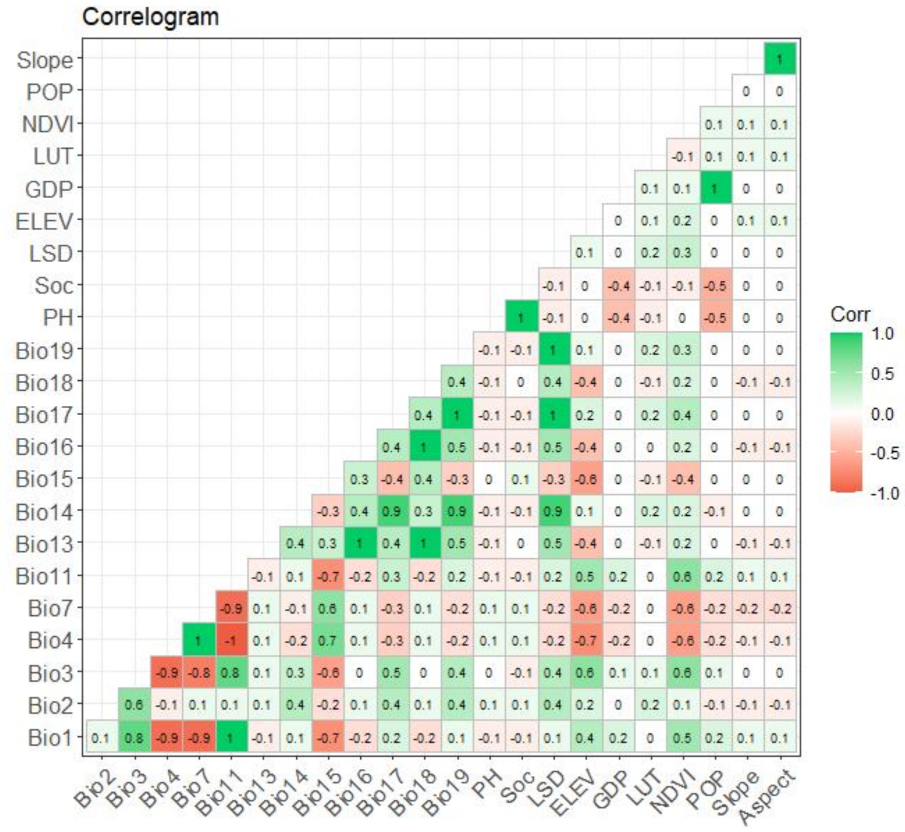

Table S3. Summary of collected Dermacentor nuttalli ticks in Inner Mongolia

Tick Species	Collection region	Collection source	Physiological status	Number of ticks
D. nuttalli	Bayannur	Sheep / Goat	Unfed adult	3542
D. nuttalli	Chifeng	Cattle / Sheep / Goat/ Camel	Unfed adult	1078
D. nuttalli	Ordos	Cattle / Sheep / Goat	Unfed adult	1202
D. nuttalli	Baotou	Cattle / Sheep	Unfed adult	215
D. nuttalli	Xiligol	Cattle / Sheep / Horse	Unfed adult	2138
D. nuttalli	Ulanqab	Sheep	Unfed adult	31
D. nuttalli	Hulunbuir	Sheep / Vegetation	Unfed adult	276
D. nuttalli	Wuhai	Sheep	Unfed adult	30
		Total		8512

Table S4 Specific PCR primer design

Target group	Target gene	Primer	Sequences (5'→3')	Fragment length/bp	Reaction temperature/℃
SFGR	*gltA*	*gltA*-F	CGAACTTACCGCTATTAGAATG	581	55
		gltA-R	CTTTAAGAGCGATAGCTTCAAG		
Ehrlichia	*P28/Omp*1	conP28-F1	AT[C/T]AGTG[G/C]AAA[A/G]TA[T/C][A/G]T[G/A]CCAA	700	55
		conP28-R1	CAATGG[A/G][T/A]GG[T/C]CC[A/C]AGA[A/G]TAG		
		conP28-F2	TTA[G/A]AA[A/G]G[C/T]AAA[C/T]CT[T/G]CCTCC	300	55
		conP28-R2	TTCC[T/C]TG[A/G]TA[A/G]G[A/C]AA[T/G]TTTAGG		
Anaplasma	*msp*2	p3726-F1	GCTAAGGAGTTAGCTTATGA	396	55
		p3761-R1	CTGCTCT[T/G]GCCAA(AG)ACCTC		
		p4183-F2	CAATAGT[C/T]TTAGCTAGTAACC	521	55
		p4257-R2	AGAAGATCATAACAAGCATTG		
Babesia	18SrRNA	BC-18SF402	GTAATTGGAATGATGGCGACTTAA	95	57
		BC-18SR496	CGCTATTGGAGCTGGAATTACC		
Borrelia	*flaB*	*flaB*-F1	TCTCACCGTTCTCTAAAGTTCAAC	793	50
		flaB-R1	CTGAATTCGGTTTCATATTTGCC		

Supplementary Methods

Risk analysis of Rickettsiae occurrence

The records for *Rickettsiae* were stored in.xls format and imported into ArcMap 10.2. They were then converted to the Shapefile format and related to the WGS 1984 projection coordinate system. The Maxent model was applied to identify areas in Inner Mongolia with suitable climatic conditions for the occurrence of *Rickettsiae*. Considering factors related to vectors and hosts, a risk analysis of *Rickettsiae* occurrence was conducted. A *Rickettsiae* risk map was produced to reveal the high-risk areas in Inner Mongolia for epidemics. The technical route was as follows:

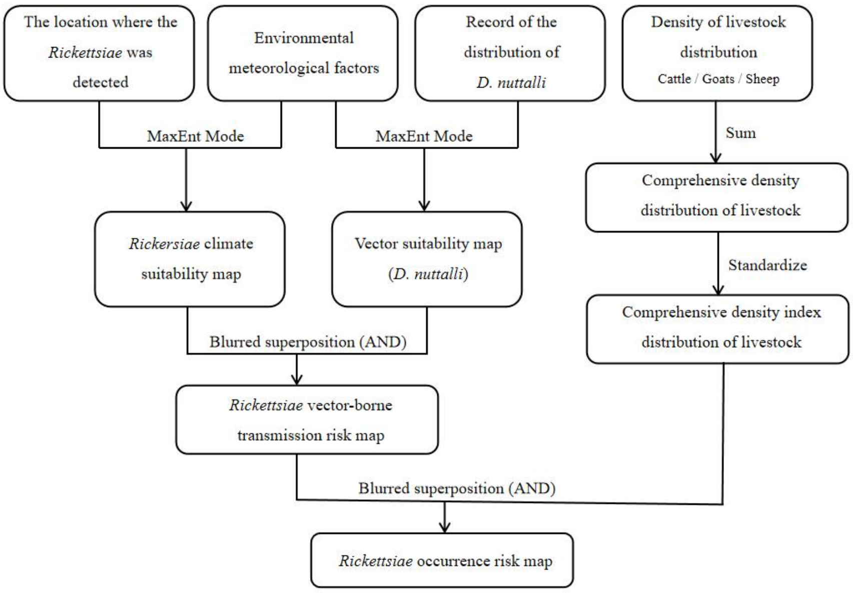

Supplementary Figure 2. Flow chart of the risk analysis of *Rickettsiae* occurrence

Using the resampling and mask extraction tools in ArcGIS, a map of the density distribution of livestock (cattle, goats, and sheep) in Inner Mongolia was extracted. The "Raster Calculator" tool under "Spatial Analyst Tools" in ArcToolbox of ArcMap 10.2 was used to combine these data, resulting in a comprehensive map of the livestock density distribution. Subsequently, it was standardized according to the method of (Tran et al., 2016), with pixel values representing the density of livestock (individuals per square kilometer) transformed through standardization into pixel values ranging from 0 to 1. Thus, the map has the same range of values as in the maps of *Rickettsiae* climate suitability and vector distribution, enabling further overlay analyses.

To assess the risk of *Rickettsiae* occurrence, a spatial overlay analysis was first conducted within the ArcMap 10.2 platform by integrating "*Rickettsiae* Climate Suitability" with "Vector Distribution" results. The specific operation was performed using the Fuzzy Overlay tool, with the overlay type set to "AND" to generate a "*Rickettsiae* Vector-Borne Risk Map." This intermediate output was then subjected to a secondary fuzzy overlay analysis with the comprehensive density index distribution of livestock, resulting in the final *Rickettsiae* occurrence risk map." The high-risk zones identified in the final output represent areas where suitable climatic conditions, vector presence probability, and a high density of susceptible animals spatially coincide, indicating a significantly elevated risk of disease outbreak.

Risk analysis of Rickettsiae transmission

The primary anthropogenic drivers facilitating the large-scale spatial dissemination of infectious diseases are transportation and trade activities. To assess the transmission risk of *Rickettsiae*, distribution data for highways and livestock trading markets within Inner Mongolia were compiled. Highway vector data (in Shapefile format) were obtained from the National Fundamental Geographic Information System. Distribution data for livestock trading markets were acquired by calling the Amap (Gaode) Map API to crawl Point-of-Interest (POI) data. After screening and validation, 264 clearly named livestock trading markets were identified and included in the analysis. Within the ArcMap 10.2 platform, the Kernel Density tool available in ArcToolbox was employed to calculate the spatial distribution density of transportation and trade factors. The resulting density raster data were then processed using the standardization method described previously, ultimately yielding a density index distribution map for each factor. Finally, the distribution maps were superimposed to obtain the traffic and trade index distribution map.

To assess the comprehensive regional distribution of host density, we obtained species occurrence point data for rodents from the Global Biodiversity Information Facility (https://www.gbif.org/) database and utilized a kernel density analysis tool to generate a spatial density distribution raster map. Subsequently, the spatial distribution data for rodent density, livestock density, and human population density were standardized separately to eliminate dimensional discrepancies. The Analytic Hierarchy Process (AHP) was used to determine the relative weights of each host factor, and a weighted overlay analysis was conducted to integrate these into a comprehensive host density index distribution dataset. The technical route was as follows:

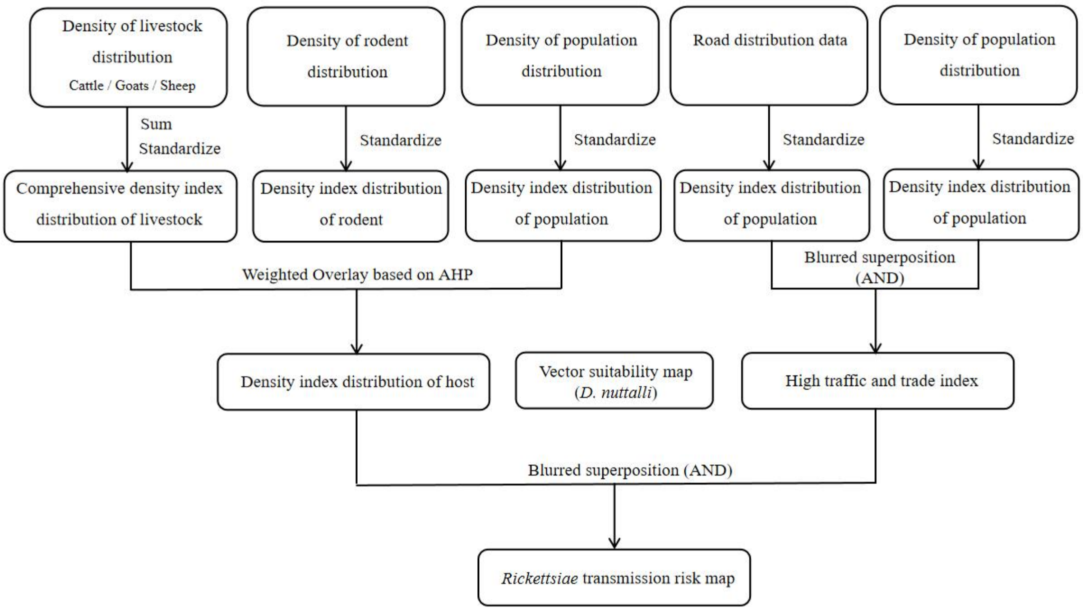

Supplementary Figure 3. Flow chart of the risk analysis of *Rickettsiae* transmission

Analytic hierarchy process (AHP) assessment system

The AHP, proposed by American operations researcher Satty, is a decision-making method that divides complex problems into interrelated and ordered hierarchies. This approach involves pairwise comparisons of the relative importance

of elements in the same hierarchy based on expert opinions and objective judgments, followed by qualitative and quantitative analyses (SAATY et al., 1994). The steps for the hierarchical analysis were as follows:

Table S5. AHP assessment system of *Rickettsiae* transmission risk

Target layer A	Criterion layer B	Indicator layer C
Rickettsiae transmission risk assessment	Host factors B_1	Rodent density C_1
		Livestock density C_2
		Population density C_3
	Transportation and trade factors B_2	Road density C_4
		Livestock trading markets density C_5
	Vector factors B_3	Vector distribution C_6

Table S6. AHP pairwise comparison matrix (A-B)

A-B	Host factors B_1	Transportation and trade factors B_2	Vector factors B_3	Weight
Host factors B_1	1	3/2	3/5	0.3
Transportation and trade factors B_2	2/3	1	2/5	0.2
Vector factors B_3	5/3	5/2	1	0.5

Table S7. AHP pairwise comparison matrix (B_1-C)

B_1-C	Rodent density C_1	Livestock density C_2	Population density C_3	Weight
Rodent density C_1	1	1	9/2	0.45
Livestock density C_2	1	1	9/2	0.45
Population density C_3	2/9	2/9	1	0.1

Consistency test was conducted: For matrix A-B, $\lambda_{max} = 3.0000$, $CI = 0$, $CR = 0 < 0.1$, indicating that the matrix passed the consistency test. For matrix B_1-C, $\lambda_{max} = 3.0000$, $CI = 0$, $CR = 0 < 0.1$, demonstrating indicating good consistency.

Table S8. Evaluation of optimization results of r-package ENMeval

Parameter settings	Regularization multiplier, RM	Featurecombination, FC	Avg. diff. AUC	delta_AICc	Mean.OR_5
Default	1	LQHP	0.782	87.0758	0.15
Optimized	2	QTH	0.811	0	0.025

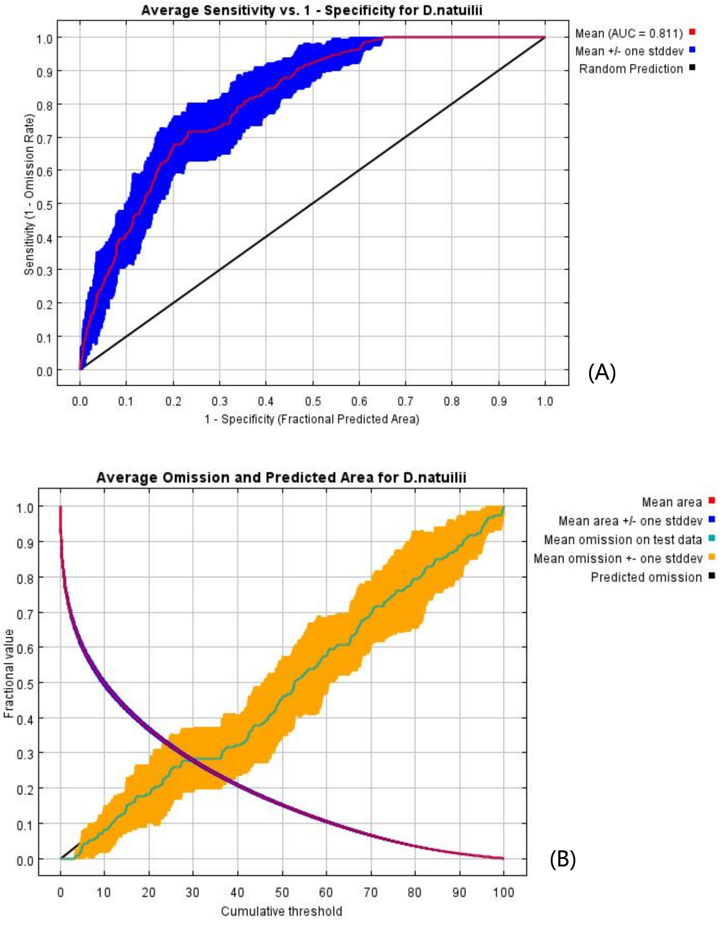

Figure S4. Model prediction accuracy analysis

Table S9. Analysis of contribution rate and importance of main environmental variables

Variable	Explanation	Percent contribution (%)	Permutation importance (%)
Bio13	Precipitation of the wettest month (mm)	56.6	50.5
Bio2	Mean monthly temperature range (℃)	28.6	7.9
LSD	Livestock density	4.1	6.7
NDVI	Normalized differential vegetation index	3.9	5.4
Bio3	Isothermality	3.8	14.7
Bio1	Mean annual temperature (℃)	1.8	10.5
ELEV	Elevation (m)	1.2	4.3

Figure S5. Binary habitat suitability map (A). Continuous suitability map (B).

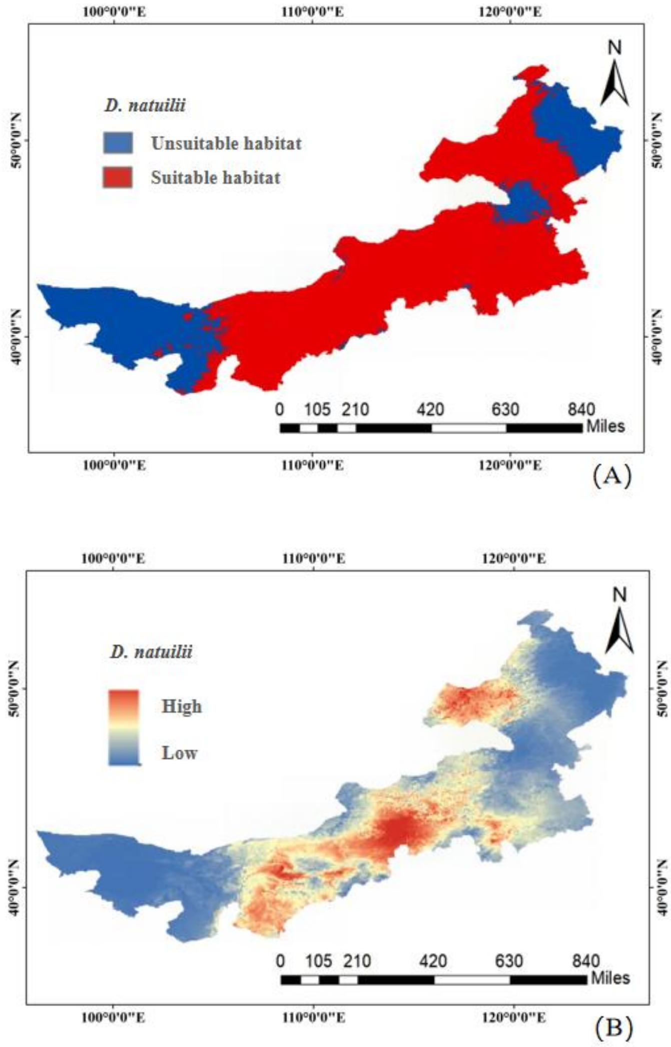

Figure S6. Positive correlations between Elevation, Soil organic content, Precipitation seasonality and the distribution of *Rickettsiae* risk in the stage 1 model.

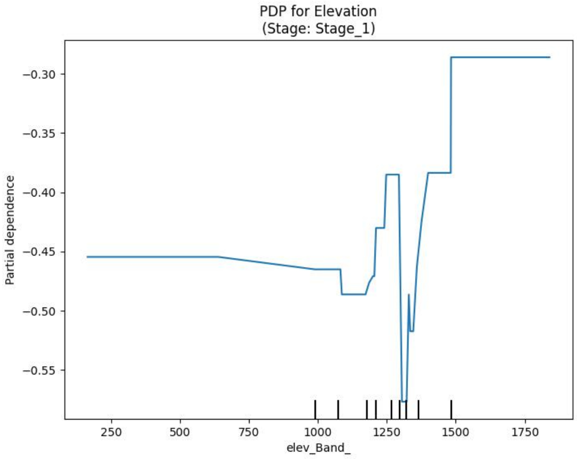

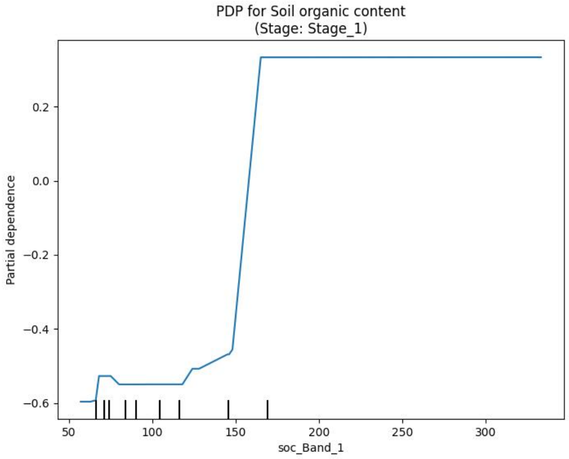

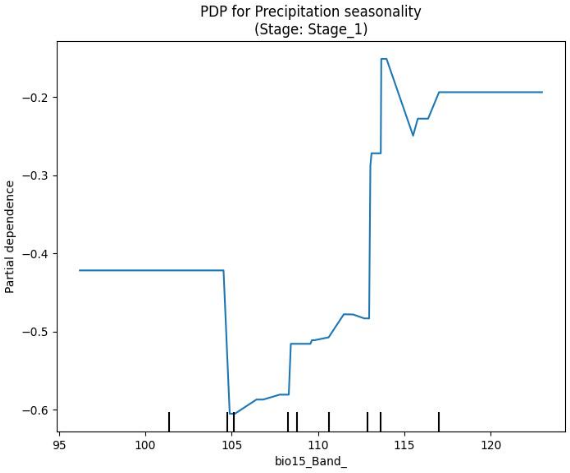

Figure S7. SHAP plots of the contribution of each variable in the stage 1 model.

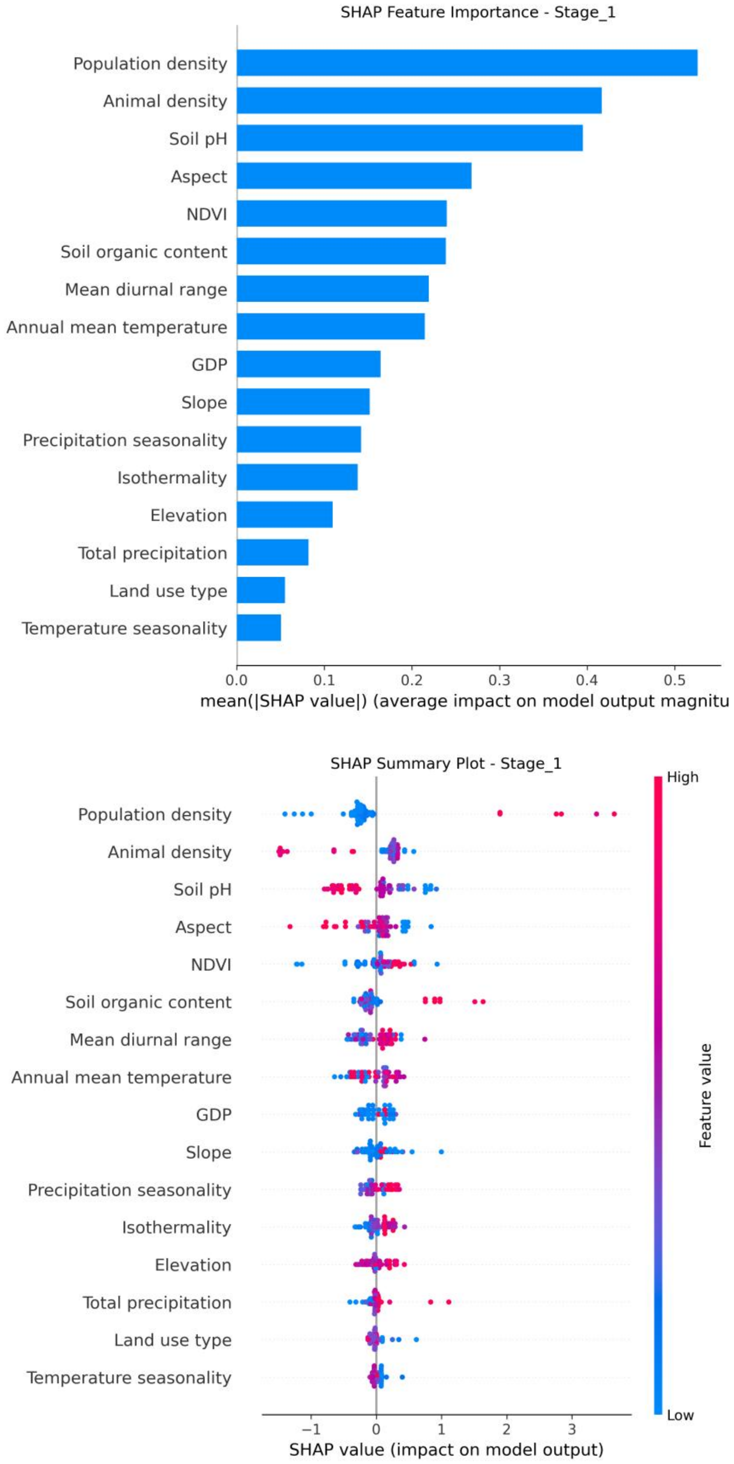

Figure S8. Negative correlations between Temperature seasonality, Isothermality, Total precipitation, and the distribution of *Rickettsiae* risk in the stage 2 model.

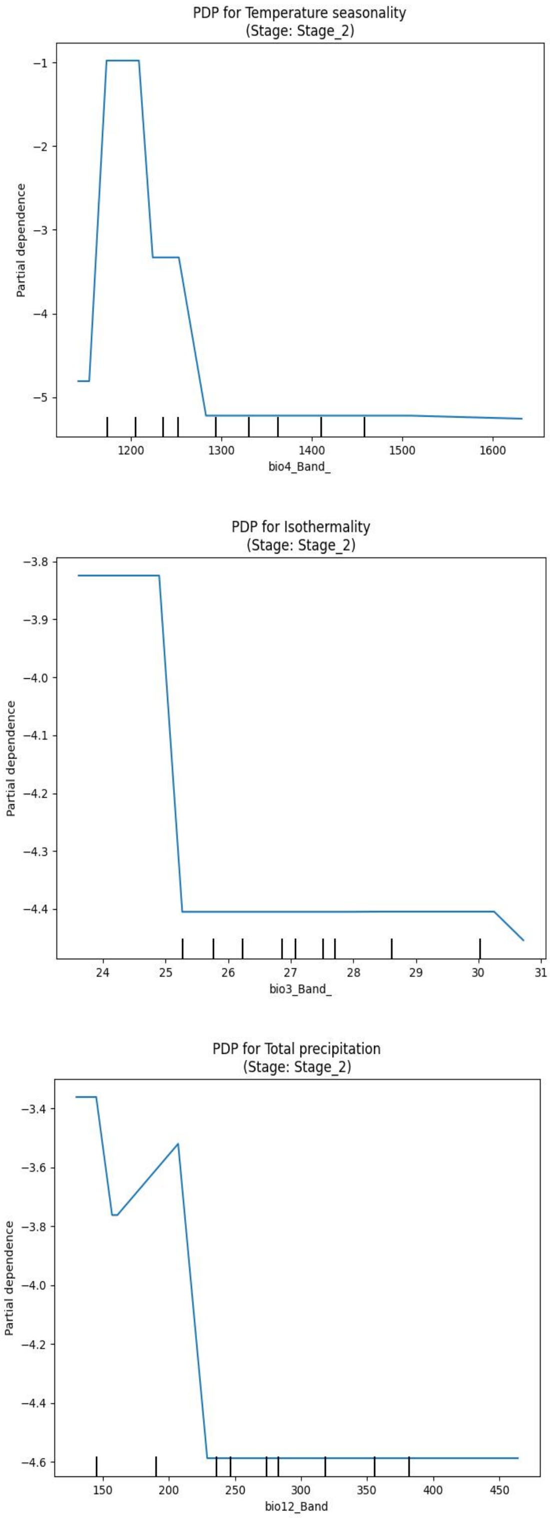

Figure S9. SHAP plots of the contribution of each variable in the stage 2 model.

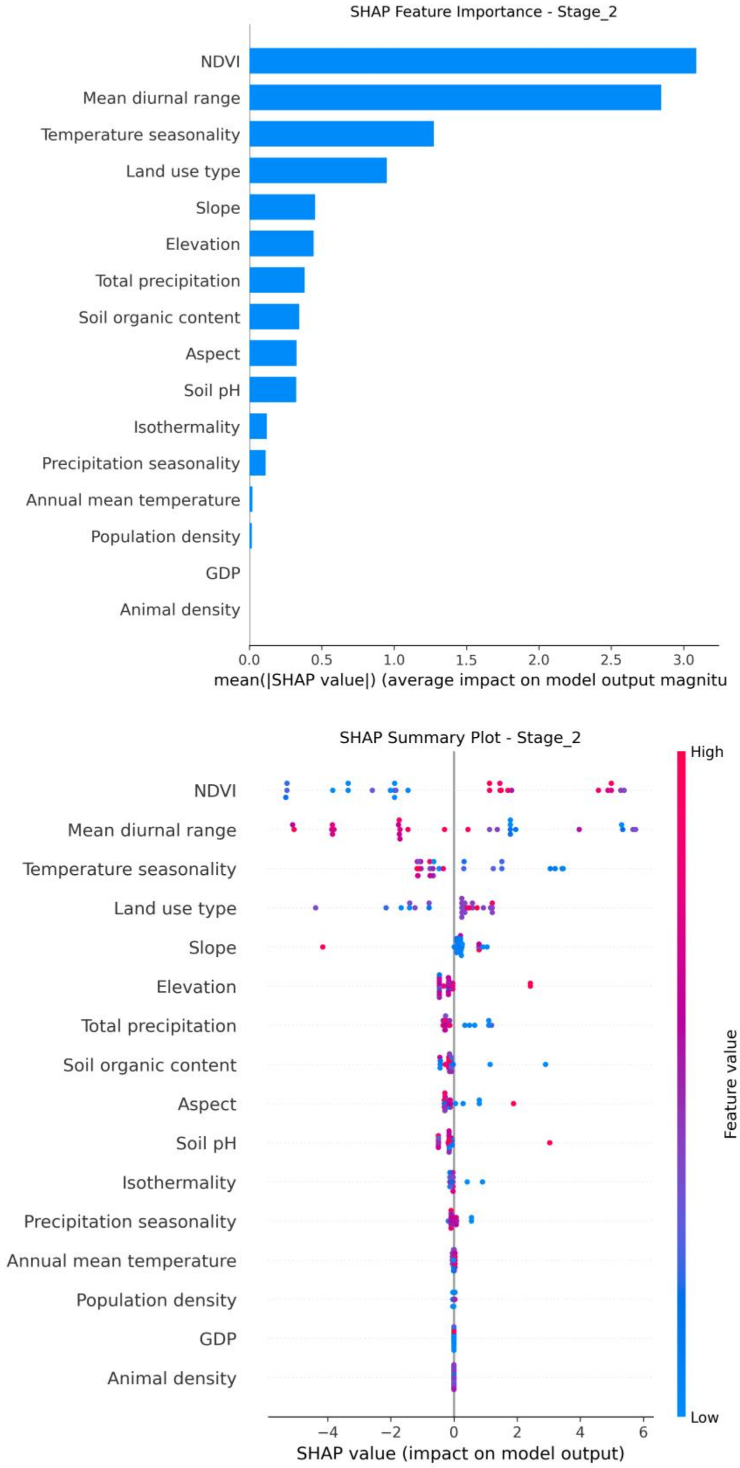

Supplementary Table S10. Performance evaluation of binary BRT model. Use the best cut-off point when the Youden index is the largest to classify the predicted value and calculate the evaluation indicators of the model.

Model	Cutoff	Sensitivity	Specificity	Accuracy	F1 score
Stage 1	0.47	0.46	0.93	0.74	0.59
Stage 2	0.49	0.85	1.0	0.96	0.92

Figure S10. Host density index

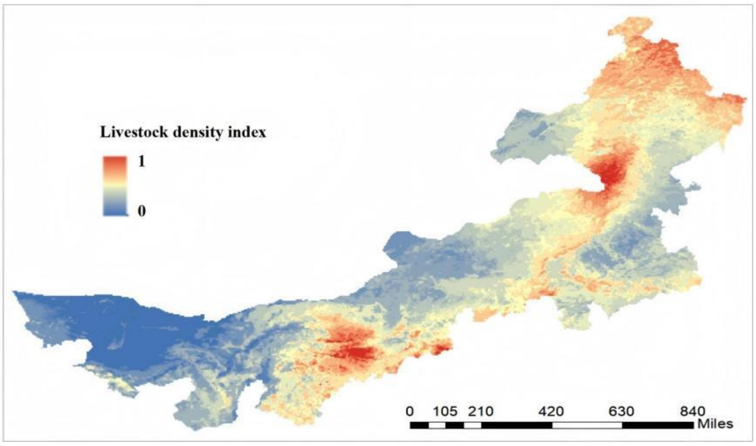

（A）Livestock density index distribution map

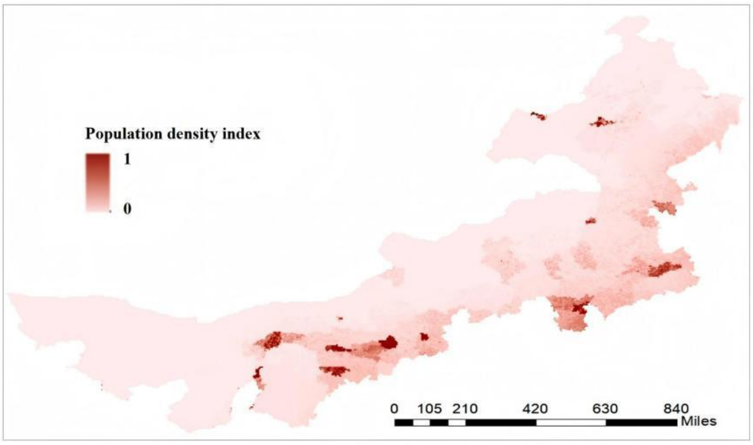

（B）Pouulation density index distribution map

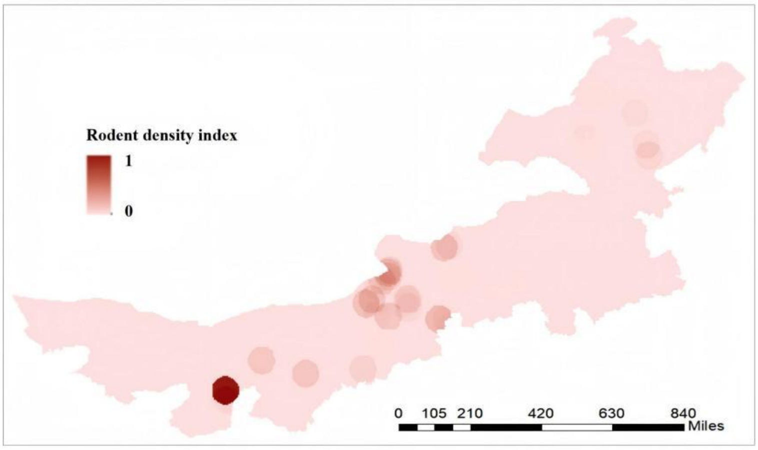

（C）Rodent density index distribution map

Figure S11. Transportation and trade composite index

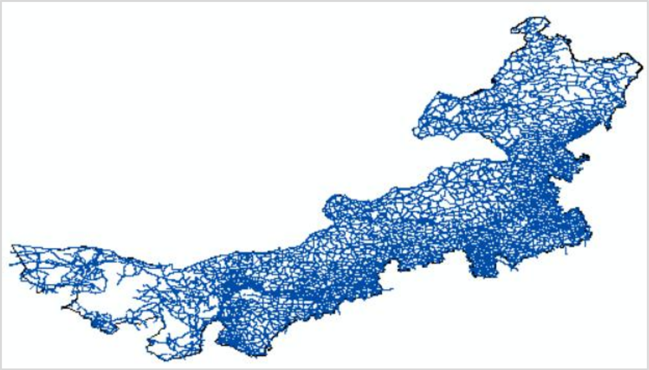

（A）Road networks distribution in Inner Mongolia

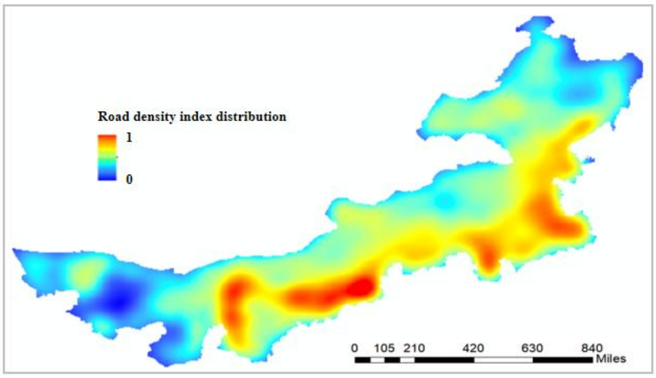

（B）Road density index distribution map

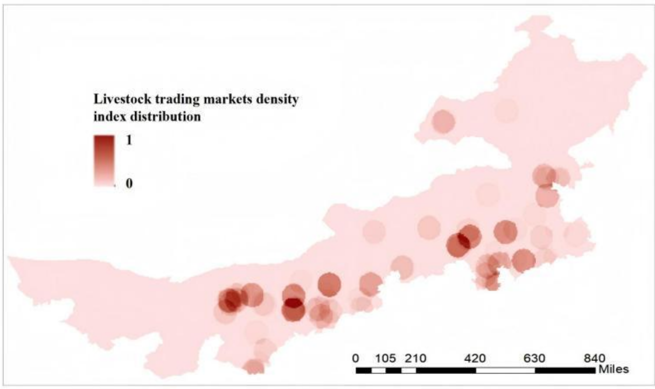

（C）Livestock trading markets distribution map

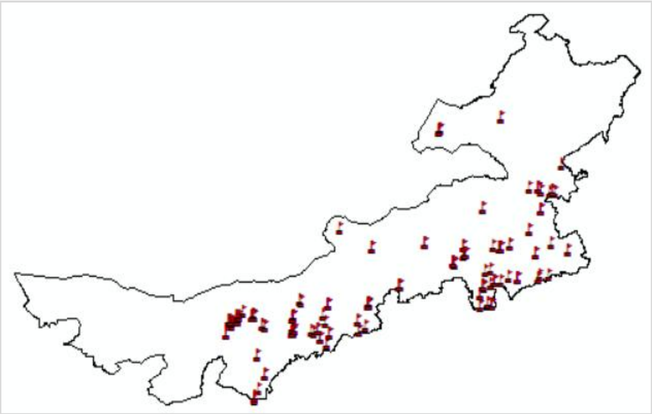

（D）Livestock trading markets density index distribution map
